# Improving error-correcting capability in DNA digital storage via soft-decision decoding

**DOI:** 10.1093/nsr/nwad229

**Published:** 2023-09-02

**Authors:** Lulu Ding, Shigang Wu, Zhihao Hou, Alun Li, Yaping Xu, Hu Feng, Weihua Pan, Jue Ruan

**Affiliations:** Shenzhen Branch, Guangdong Laboratory of Lingnan Modern Agriculture, Genome Analysis Laboratory of the Ministry of Agriculture and Rural Affairs, Agricultural Genomics Institute at Shenzhen, Chinese Academy of Agricultural Sciences, Shenzhen518120, China; Shenzhen Branch, Guangdong Laboratory of Lingnan Modern Agriculture, Genome Analysis Laboratory of the Ministry of Agriculture and Rural Affairs, Agricultural Genomics Institute at Shenzhen, Chinese Academy of Agricultural Sciences, Shenzhen518120, China; Shenzhen Branch, Guangdong Laboratory of Lingnan Modern Agriculture, Genome Analysis Laboratory of the Ministry of Agriculture and Rural Affairs, Agricultural Genomics Institute at Shenzhen, Chinese Academy of Agricultural Sciences, Shenzhen518120, China; Guangdong Provincial Key Laboratory of Plant Molecular Breeding, State Key Laboratory for Conservation and Utilization of Subtropical Agro-Bioresources, South China Agricultural University, Guangzhou510642, China; Shenzhen Branch, Guangdong Laboratory of Lingnan Modern Agriculture, Genome Analysis Laboratory of the Ministry of Agriculture and Rural Affairs, Agricultural Genomics Institute at Shenzhen, Chinese Academy of Agricultural Sciences, Shenzhen518120, China; Shenzhen Branch, Guangdong Laboratory of Lingnan Modern Agriculture, Genome Analysis Laboratory of the Ministry of Agriculture and Rural Affairs, Agricultural Genomics Institute at Shenzhen, Chinese Academy of Agricultural Sciences, Shenzhen518120, China; Shenzhen Branch, Guangdong Laboratory of Lingnan Modern Agriculture, Genome Analysis Laboratory of the Ministry of Agriculture and Rural Affairs, Agricultural Genomics Institute at Shenzhen, Chinese Academy of Agricultural Sciences, Shenzhen518120, China; Shenzhen Branch, Guangdong Laboratory of Lingnan Modern Agriculture, Genome Analysis Laboratory of the Ministry of Agriculture and Rural Affairs, Agricultural Genomics Institute at Shenzhen, Chinese Academy of Agricultural Sciences, Shenzhen518120, China; Shenzhen Branch, Guangdong Laboratory of Lingnan Modern Agriculture, Genome Analysis Laboratory of the Ministry of Agriculture and Rural Affairs, Agricultural Genomics Institute at Shenzhen, Chinese Academy of Agricultural Sciences, Shenzhen518120, China

**Keywords:** DNA digital storage (DDS), error-correcting code (ECC), soft-decision decoding, error-correcting capability, storage volume

## Abstract

Error-correcting codes (ECCs) employed in the state-of-the-art DNA digital storage (DDS) systems suffer from a trade-off between error-correcting capability and the proportion of redundancy. To address this issue, in this study, we introduce soft-decision decoding approach into DDS by proposing a DNA-specific error prediction model and a series of novel strategies. We demonstrate the effectiveness of our approach through a proof-of-concept DDS system based on Reed-Solomon (RS) code, named as Derrick. Derrick shows significant improvement in error-correcting capability without involving additional redundancy in both *in vitro* and *in silico* experiments, using various sequencing technologies such as Illumina, PacBio and Oxford Nanopore Technology (ONT). Notably, *in vitro* experiments using ONT sequencing at a depth of 7× reveal that Derrick, compared with the traditional hard-decision decoding strategy, doubles the error-correcting capability of RS code, decreases the proportion of matrices with decoding-failure by 229-fold, and amplifies the potential maximum storage volume by impressive 32 388-fold. Also, Derrick surpasses ‘state-of-the-art’ DDS systems by comprehensively considering the information density and the minimum sequencing depth required for complete information recovery. Crucially, the soft-decision decoding strategy and key steps of Derrick are generalizable to other ECCs’ decoding algorithms.

## Teaser

Though existing inspiring encoding strategies, the information density of DNA digital storage is limited by hard-decision decoding. By exploiting error bias in sequencing and alignment, soft-decision decoding with novel strategies doubles the error-correcting capability of RS code.

## INTRODUCTION

As digital data production exponentially grows and mainstream magnetic, optical and solid-state storage approaches their density limits [1], DNA has been seen as an attractive alternative for archival storage [2] due to its advantages in information density [3,4] and durability [5–7]. DNA digital storage (DDS) systems encode information into nucleotide sequences, and then synthesize, replicate and store DNA molecules correspondingly. To restore the information, DDS sequences the DNA molecules, assembles the reads (DNA fragments from sequencing) into consensus and carries out decoding. In the whole process, there are many steps such as synthesis, replication, storage and sequencing which may induce random and systematic errors, thus proper error-correction strategies need to be used within the decoding procedure.

Nearly all error-correction methods rely on information redundancy to ensure the correctness of the restored information, including physical redundancy and logical redundancy [1,8,9]. Traditional physical redundancy-based methods explicitly copy the DNA molecules for one or more times and expect every piece of information to correctly appear in the majority of copies while decoding [3,10]. Although the physical redundancy is able to solve random errors, the systematic errors such as sequence missing caused by polymerase chain reaction (PCR) stochastic bias or synthesis bias [9], as well as strand breaks, rearrangements and indels from PCR amplification and long-term storage [11] are usually beyond its capability. Therefore, the state-of-the-art DDS systems mostly use logical redundancy in the form of error-correcting code (ECC) [12] instead.

ECC calculates and adds the redundancy [13] which contains scattered information from every unit of the original data block by applying a series of mathematical transformations when encoding and thus is able to detect and correct a limited number of errors when decoding by finding a feasible solution in inverse transformation. Previous studies have utilized various ECC methods to improve error-correcting capability. For instance, Grass *et al.* applied ECC in the area of DDS for the first time [14]. They used Reed-Solomon (RS) code in a concatenated way, which was able to correct not only single-nucleotide errors, but also the loss of oligos. Erlich *et al.* combined RS code with a type of ECC approaching Shannon capacity theoretically, called Fountain code, for high information density [4]. Press *et al.* developed a new ECC system called HEDGES which worked well for correcting insertion and deletion errors [15]. Although these efforts improve the performance of ECC in DDS to some extent, the state-of-the-art methods still suffer from a trade-off between error-correcting capability and redundancy proportion. In other words, solving more errors needs a higher proportion of redundancy which leads to lower information density. For example, in the experiments of Press *et al.* [15], an error rate of approximately 1% corresponds to an information density of 1.2 bits per nucleotide, while an error rate of approximately 3% results in an information density of 1 bit per nucleotide.

In this paper, we introduce a novel idea for generally improving the error-correcting capability of ECC without increasing the proportion of redundancy. More specifically, we exploit the uneven distribution of errors in DNA sequences and leverage the error-related key information such as error positions and true values that can be predicted based on the detected error-enriched patterns in the consensus sequence, and this type of information provides opportunities for ECC to address blocks with error counts that exceed the original capability of ECC. As a proof of concept, we develop a new ECC system called Derrick (de-error-rick), utilizing RS code [16] which is the most commonly used ECC for DDS by introducing the soft-decision decoding strategy [17,18] from communication engineering. Due to the difference between the DNA sequences and the binary sequences, the soft-decision decoding strategies used in communication cannot be applied in the area of DDS directly. As mentioned by a previous review paper [1], ‘unlike other storage channels, which have only substitution errors, DNA channels can also manifest base insertions and deletions, which makes coding more challenging.’ To address this issue, the error model was designed for describing different types of errors including substitutions, insertions and deletions, and a series of novel strategies were proposed in Derrick.

The performance of Derrick was verified through both *in silico* and *in vitro* tests. In the *in vitro* experiments with Oxford Nanopore Technology (ONT) sequencing of depths ranging from 4× to 10×, compared with traditional hard-decision decoding, Derrick improved the number of solvable errors of RS code by up to 2-fold, reduced the proportion of matrices with decoding-failure by up to 229-fold, and increased the storage volumes by up to 91 504-fold. With Illumina sequencing, Derrick demonstrated the potential to improve maximum storage volume to Brontobyte-scale with the same calculation method as in previous work [15]. Additionally, Derrick surpasses the ‘state-of-the-art’ DDS systems in error-correcting capability by comprehensively considering the information density and the minimum sequencing depth required for complete information recovery, although it is just a proof-of-concept system.

## RESULTS

### Overview of Derrick algorithm

**Figure 1.**
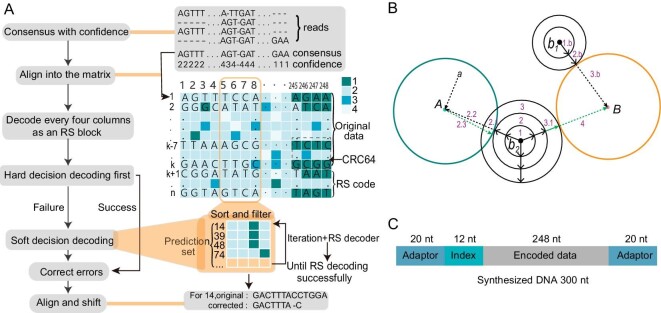
Schematic diagram of Derrick decoding algorithm and soft-decision decoding strategy with sequence composition of synthesized DNA molecules. (A) The Pipeline of Derrick decoding algorithm. The left panel provides a literary description of the steps of Derrick algorithm, while the right panel depicts the consensus building, error prediction, soft-decision strategy and shifting steps. Initially, the consensus sequence is generated from the aligned reads and the confidence score of each nucleotide is calculated. Subsequently, the consensus sequence is cut and reassembled into matrices where the yellow frame on the matrix represents an RS block and different colors of the matrix indicate different confidence scores. For each block, the algorithm first tries the hard-decision decoding approach. If this fails, it employs the soft-decision decoding strategy, selecting the positions with the lowest confidence scores as potential error positions and adding them to the prediction set. The prediction set is then used for iterative soft-decision decoding until the block is successfully corrected. After each block is decoded, the algorithm adjusts the erroneous subsequences in the matrix based on the realignments between the original and corrected sequences to reduce errors in subsequent blocks. (B) Schematic diagram of RS soft-decision decoding strategy. Three RS codes, *a, b*_1_ and *b*_2_, are to be decoded (corrected). *A* and *B* are two candidate solutions that the RS codes (*a, b*_1_ and *b*_2_) may be decoded (corrected) into. The blue and orange circles represent the error-correcting capability of the hard-decision strategy for *A* and *B*. In Derrick, since *a* is within the decoding capability of *A* (inside blue circle), it is corrected into *A* by hard-decision strategy. For *b*_1_, Derrick carries out soft-decision decoding by gradually increasing the black circle representing iteratively searching larger subsets of the prediction set until it (the second black circle) reaches the capability (orange circle) of *B* and is corrected into *B*. Similarly, *b*_2_ is corrected into *A* by the soft-decision strategy in Derrick after reaching the capability (blue circle) of *A*. Although *A* is the closest solution to *b*_2_, the correct solution is *B* (the second closest solution). Therefore, CRC64 shows the mistake, and the algorithm backtracks and recorrects *b*_2_ to *B* by continuing to search larger subsets of the prediction set (increasing black circle) until it reaches the capability (orange circle) of *B* to correct *b2* into *B*. (C) Sequence composition of each synthesized DNA molecule, where ‘nt’ represents nucleotides.

First of all, we introduce the principle of soft-decision strategy which is the key idea in Derrick (Fig. 1A). In general, RS decoder corrects the errors by solving a system of equations [19–21], in which each error is represented as one or more unknown variables. More specifically, RS decoder classifies errors into two types: (1) ‘error’ with both position and true value as unknown variables, and (2) ‘erasure’ with position known and only true value as unknown variable. In contrast to the traditional hard-decision strategy used in existing ECC technologies for DDS channel, which treats all errors as ‘error’, the soft-decision decoding strategy [18] reduces the number of variables for data blocks by converting ‘errors’ to ‘erasures’ (each reduces one variable) or removing ‘errors’ entirely (reducing two variables per removal). This is done using the priorly predicted error positions and true values based on an error prediction model, as outlined in Supplementary Note 1. We develop a general prediction model by calculating nucleotide confidences from the numbers of supporting reads during consensus building (Fig. 1A). This model enables the implementation of soft-decision decoding to correct data blocks that cannot be corrected by hard-decision decoding.

Although the error positions can easily be predicted directly from nucleotide confidences, predicting true values on DNA sequences with four characters (A, T, C, G) and multiple error types such as substitution, insertion and deletion is more challenging. To bypass this problem, when building consensus DNA sequences from sequencing reads, Derrick generates comparatively loose and long alignments by adding as many as possible spaces into reads, converting other types of errors into insertions and enabling the easy correction of errors by removal. As illustrated in Fig. 1A, we then proposed the Derrick algorithm for error-correction decoding, which comprises a few key points as follows.

Due to the imperfection of error prediction, the set of predicted error positions and true values inevitably contains false-positives. However, RS decoder can judge the correctness of the predictions participating in soft-decision process, as any incorrect prediction can introduce more errors making the soft-decision strategy futile and leading to decoding failure. Thus, Derrick sorts the predicted set by erroneous possibility based on confidence level, and then uses an iterative algorithm to attempt subsets from the smallest to the largest one-by-one (Fig. S1) until RS correction succeeds or all subsets have been tried (soft-decision strategy failed) (details in Supplementary Note 1). Larger prediction sets offer greater chances of reducing enough variables to successfully solve blocks with errors exceeding RS original capability, but require higher computational costs of searching feasible subsets. Thus, with this trade-off, Derrick improves the error-correcting capability of RS code by sacrificing computation efficiency to some extent, and is capable of solving any data block with any number of errors with unlimited computational resources. In practice, Derrick limits computational time for each data block using a threshold and gives up blocks beyond this threshold.

As indels shift the reading frames of the sequences causing errors, not only in the affected blocks but also all downstream blocks, the decoding process of the related matrices may fail due to the timeouts caused by excessive errors. To eliminate these errors in the downstream blocks, a shift algorithm was designed to realign and shift the rows of the matrix according to the new corrected values after each RS correction (Supplementary Note 1, Fig. S2).

RS decoder corrects blocks with errors by finding a solution to the system of equations that is closest to the original values in space, even if there are multiple solutions available [22]. However, this may not always guarantee that the true value is obtained, and some corrected blocks may still be wrong [23] (Fig. 1B). This issue is not unique to RS decoding and applies to other ECCs as well. To address this, Derrick uses CRC64 [24,25] code to verify the correctness of the whole matrix (a matrix has 62 columns representing 62 continuous blocks and 255 rows and each entry contains 4 nucleotides; Fig. 1A) after all blocks of it have been corrected by RS decoder. If any error is detected, Derrick uses greedy strategy-based algorithms (see backward-searching algorithm and forward-searching algorithm in Supplementary Note 1) to backtrack and recorrect the blocks.

### Performance of the soft-decision decoding strategy and Derrick algorithm

To validate and benchmark the Derrick decoding algorithm, we conducted both *in vitro* and *in silico* tests. Although there are a few public large-scale DDS real datasets available for testing, they are limited to single sequencing technology or single code rate. Therefore, we generated a new Megabyte-scale real dataset from *in vitro* experiments consisting of 21 sub-datasets of Illumina sequencing and 16 sub-datasets of ONT sequencing with varying code rates (0.83, 0.92 and 0.95) and sequencing depths (from 2× to 10×). This dataset is currently the most complete and publicly available DDS real dataset. The dataset was generated by concatenating DNA sequences from an *E. coli* genome and 18 COVID-19 genomes (Table S1A), followed by Derrick pre-processing steps such as compression, randomization, redundancy addition, index and primer addition, synthesizing, sequencing, and subsampling (details in Supplementary Note 2). All *in vitro* experiments were conducted on this real dataset.

**Figure 2.**
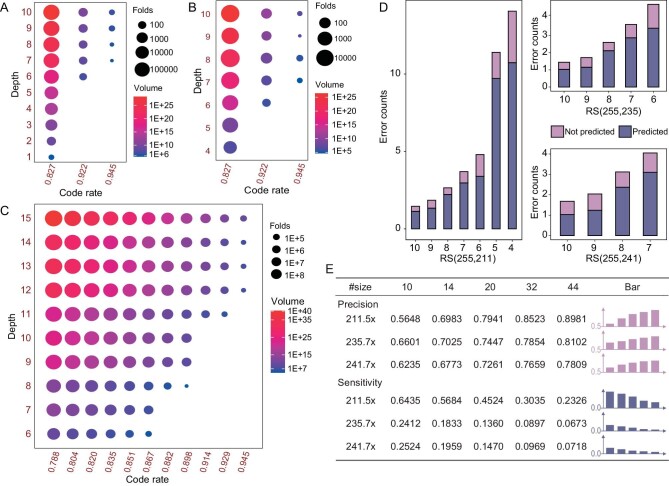
Results of Derrick decoding algorithm and soft-decision strategy via *in vitro* and *in silico* experiments. (A) Improvement of Derrick compared with hard-decision decoding on *in vitro* ONT datasets. The horizontal and vertical axis represent the code rate and sequencing depth, respectively. The size of the circle represents the folds of improvement which is measured by the ratio of the probability of an uncorrectable error calculated using the soft-decision strategy versus that calculated using the hard-decision strategy. The color shading of the circle represents the achievable storage volumes of Derrick. (B) Improvement of Derrick compared with hard-decision decoding on *in vitro* Illumina datasets. The meanings of horizontal axis, vertical axis, size and color of the circle are all same as (A). (C) Improvement of the soft-decision strategy compared with the hard-decision one on PacBio CLR simulated datasets. The meanings of horizontal axis, vertical axis, size and color of the circle are all same as (A). (D) Error prediction performance of the soft-decision strategy on *in vitro* ONT datasets. The horizontal and vertical axis represent the sequencing depth and the number of errors, respectively. The color indicates the average number of successfully predicted errors (represented by the dark color) and the number of errors failed to predict (represented by the shallow color) for each block. (E) Comparison of the prediction performance of Derrick with different sizes of the prediction set, tested via *in vitro* ONT experiments. The bar plot of precision shows a tendency of improvement with the increasing size of the prediction set, with the y-axis starting from 0.5. Conversely, the bar plot of sensitivity shows a tendency of decrease with the increasing size of the prediction set, with the y-axis starting from 0. The ‘#size’ represents the size of the prediction set; ‘RS211.5× ’ represents RS (255, 211) and sequencing depth 5×; ‘RS235.7× ’ and ‘RS241.7× ’ represent similarly as ‘RS211.5× ’.

First of all, the error-correcting capability of soft-decision decoding is demonstrated by comparing the number of solvable errors with the traditional hard-decision one. As shown in Table 1, the *in vitro* experiments with ONT sequencing show that the soft-decision strategy was able to solve the blocks beyond the capability of the hard-decision strategy by correcting 1 to 8 more errors each. On the two tests (sequencing depths 6× and 7×, code rate 0.95) with the best results, it solved 14 errors which is twice that of hard-decision decoding. The performance of Derrick is also evaluated on a per-matrix basis, where the proportion of failed matrices after each decoding experiment is recorded and analyzed. Table 2 shows that, on ONT real data, for the datasets that hard-decision decoding failed to decode, Derrick either successfully corrected all matrices or reduced the proportions of failed ones by 7- to 229-fold. For example, with the depth 8× and a code rate of 0.945 (RS (255, 241)), Derrick reduced the number of failed matrices from 35 (43.75%) to 0. Overall, Derrick reduced the sequencing depth required for successful decoding. AS for Illumina real datasets, the decoding results comparisons under various conditions, such as different code rates and sequencing depths, are given in Table S2, presenting a similar conclusion as ONT real data. In addition, a statistical model was constructed to evaluate the performance of Derrick by the probability of an uncorrectable error (Methods and Supplementary Note 3) with the same calculation method as previous work [15]. As shown in Fig. 2A and 2B, Derrick reduced the probability of an uncorrectable error of RS code by ∼10- to ∼500k-fold on Illumina and ONT datasets. The best improvement was achieved on an Illumina dataset with sequencing depth 10× and a code rate of 0.83, increasing the maximum storage volume from 2.77E+22 bytes to 1.39E+28 bytes, achieving Brontobyte-scale. Additionally, we conducted a performance comparison between Derrick and the ‘state-of-the-art’ DDS systems. However, due to differences in coding modes, it was not feasible to test multiple DDS systems on the same dataset. Therefore, we followed the conventions [1,4] in the area of DNA digital storage and compared the best performance statistics of different systems on their respective testing datasets. Table 3 illustrates that, by comprehensively considering the information density and the minimum sequencing depth required for fully recovering the information, Derrick performed almost the best among the DDS systems published in recent years, although it is just a proof-of-concept system.

**Table 1.** Comparison of Derrick and hard-decision decoding by the number of correctable errors on ONT real datasets.

RS (*n, k*)	RS (255, 211)	RS (255, 235)	RS (255, 241)
**(*n−k*)/2**	**22**	**10**	**7**
**4**×	**30**	–	–
**5**×	**24**	–	–
**6**×	16	**18**	**14**
**7**×	10	**17**	**14**
**8**×	7	**11**	**9**
**9**×	7	9	**8**
**10**×	6	8	6

The ‘(n−k)/2’ row shows the maximum number of correctable errors achievable through the hard-decision strategy. Here, ‘n’ and ‘k’ refer to the lengths of the entire RS code and the original information contained within, respectively; the remaining rows of the table show the maximum number of errors corrected in each test, with bold values indicating tests where the number of errors exceeds the capability of hard-decision strategy and must be resolved using soft-decision strategy. Normal values represent tests with the number of errors where the number of errors falls within the hard-decision strategy's capabilities and do not require soft-decision strategy; ‘–’ denotes tests where the number of errors exceeds the soft-decision strategy's capacity, and the result is, therefore, not considered.

**Table 2.** Performance comparison of Derrick decoding with hard-decision decoding by the number of failed matrices on ONT real datasets.

	RS(255, 211) (#Matrix = 120)		RS(255, 235) (#Matrix = 1600)		RS(255, 241) (#Matrix = 80)	
Depth	Hard	Derrick	Hard	Derrick	Hard	Derrick
4×	78	1	–	–	–	–
5×	15	0	–	–	–	–
6×	0	0	887	41	–	–
7×	0	0	229	1	71	10
8×	0	0	31	0	35	0
9×	0	0	1	0	3	0
10×	0	0	0	0	0	0

The ‘#Matrix’ displays the total number of matrices used in each decoding test; the ‘Depth’ column represents the sequencing depths; the ‘Hard’ and ‘Derrick’ columns indicate the number of failed matrices observed in tests utilizing the hard-decision decoding strategy and Derrick decoding algorithm, respectively; ‘–’ denotes tests where the number of errors exceeds the soft-decision strategy's capacity, and the result is, therefore, not considered.

**Table 3.** Comparison of information density and required sequencing depth to prior work.

Study	Data encoded	Synthesis	Error correcting	Sequencing platform	Physical redundancy	Information density (bits/bp)
Church *et al.* [3]	650 kB	Phosphoramodite	**High coverage**	Illumina	3000×	0.6
Goldman *et al.* [10]	630 kB	Phosphoramodite	**Multiply copies**	Illumina	51×	0.19
Grass *et al.* [14]	80 kB	Phosphoramodite	**RS + RS**	Illumina	372×	0.86
Bornholt *et al.* [34]	151 kB	Phosphoramodite	**Huffman code**	Illumina	128×	0.57
Blawat *et al.* [25]	22 MB	Phosphoramodite	**RS + BCH + CRC16**	Illumina	160×	0.89
Erlich *et al.* [35]	2 MB	Phosphoramodite	**Fountain + RS**	Illumina	10.5×	1.19
Yazdi *et al.* [36]	3 kB	Phosphoramodite	**MSA**	ONT	200×	1.71
Organick *et al.* [8]	33 kB	Phosphoramodite	**RS + RS**	ONT	36×	0.81
Organick *et al.* [8]	200 MB	Phosphoramodite	**RS + RS**	Illumina	5×	0.81
Lee *et al.* [37]	96 B	Enzymatic	**RS + BCH**	ONT	175×	1.57
Chandak *et al.* [38]	11 kB	Phosphoramodite	**Convolution + RS**	ONT	14×	0.56
Meiser *et al.* [6]	176 kB	Phosphoramodite	**RS + RS**	Illumina	200×	0.85
Press *et al.* [15]	2 MB	Phosphoramodite	**Hash + RS**	Illumina	50×	1.2
Weigang *et al.* [39]	37.8 kB	Phosphoramodite	**LDPC**	ONT	16.8×	1.19
Lifu *et al.* [11]	6.8 MB	Phosphoramodite	**Fountain + CRC**	Illumina	–	1.3
This work	5.2 MB	Phosphoramodite	**RS + CRC**	Illumina	4×	1.37
This work	5.2 MB	Phosphoramodite	**RS + CRC**	ONT	8×	1.56

‘–’, denotes not available.

To comprehensively evaluate the performance of Derrick on a wider variety of data with a wider range of different code rates and sequencing techniques, we prepared a larger file library of 11.7 MB in total for *in silico* tests, which contained 6 files of different types (Table S1B) such as videos, photos and executable files. The files were merged, encoded with varied code rates, and then subjected to sequencing simulation using currently popular sequencing techniques, including PacBio CLR, ONT and Illumina. More detailed, Illumina and ONT datasets were built with RS (255, 211), RS (255, 235) and RS (255, 241), and PacBio CLR datasets were with a wider range of RS codes, ranging from RS (255, 201) to RS (255, 241) with an interval of 4. The results of the simulated datasets demonstrated that, compared with hard-decision decoding, Derrick decreased the probability of an uncorrectable error by tens to tens of thousands of folds (Fig. 2C and Fig. S3B), and reduced the numbers of failed matrices from hundreds to units, in most cases to 0 (Table S3–5).

### Factors related to the performance of Derrick algorithm

The performance of the soft-decision strategy and Derrick decoding algorithm strongly depends on the accuracy of error prediction. The results show that the soft-decision strategy achieved high error-prediction accuracy of 76.7% on ONT real datasets (Fig. 2D), 72.3% on Illumina real datasets (Fig. S4) and 86.51% on ONT simulated datasets (Fig. S3A). We also found that the accuracy of error prediction is strongly related to the size of prediction set. More specifically, the sensitivity increases but the precision decreases as the prediction size increases (Fig. 2E). One step further, we recommend setting the number of predicted errors to be the same as or slightly larger than the length of redundancy (*n − k*, where *n* and *k* are the lengths of whole RS code and the original information, respectively) (Table S6) in a RS code, because theoretically *n −k * is the upper bound of RS correction capability and at most *n − k* ‘errors’ need to be changed into ‘erasures’.

Also, to study how the performance of Derrick decoding algorithm is related to the time limit of each matrix, we show the running time distributions of matrices. Supplementary Table 7 shows that, on the whole, over 96% of the matrices were completed within 10 seconds, with only 1.6% taking more than 6 000 seconds. From one perspective, the result suggests that a comparatively short time limit is sufficient for solving the majority of matrices. But from another perspective, it also implies that, to improve the number of solved matrices, the running time limit has to be increased exponentially. One potential method to solve these remaining matrices within an acceptable time is to increase the RS code size, resulting in a more evenly distributed number of errors within RS blocks and allowing an appropriate time threshold to cover more data blocks. However, due to the non-linear growth of RS correction time as code size increases, further research is needed to verify the feasibility of this idea.

To demonstrate the importance of CRC64 code [24] checking, we conducted experiments and collected statistics, presented in Supplementary Table 8. Our results demonstrate that, in the absence of CRC64 checking, approximately 1.5% of matrices would be wrongly-corrected ones unknown by users. However, by employing CRC64 checking and backtracking one or more times within the limited runtime (6000 s), we successfully recorrected 51.8% of these matrices. Additionally, we conducted experiments on ONT simulated datasets to demonstrate the importance of the shift algorithm designed for realigning the sequences (Table S9). Our results reveal that none of the matrices can be solved by Derrick without the shift algorithm, which was due to the huge number of errors caused by mis-alignments.

## DISCUSSION

Error-correcting code is indispensable in DDS for ensuring data correctness and integrity [26]. State-of-the-art DDS systems use the hard-decision decoding strategy, which is restricted to the trade-off between error-correcting capability and information density. In this study, we propose a novel soft-decision decoding strategy in DDS, and a proof-of-concept DDS system, Derrick, on RS code which is capable of improving the error-correcting capability of ECC without reducing the information density by making accurate error predictions. We verify the performance of the soft-decision strategy and Derrick across different types of sequencing technologies such as ONT, Illumina and PacBio. The experimental results show that Derrick improved the error-correcting capability of RS code by up to 2-fold over the hard-decision one, and Derrick was able to recover with 100% accuracy even on low-depth noisy sequencing data. The results of our statistical modeling also show that Derrick was able to solve more errors than traditional hard-decision decoding, reducing the probability of uncorrectable errors by several orders of magnitude. Our findings have important implications for the development and optimization of DNA digital storage systems, and can help to improve the performance of these systems.

It should be emphasized that soft-decision decoding in DDS cannot be simply adopted from the communication field. Unlike binary sequences in communication, DDS channels are composed of four characters (‘A’, ‘T’, ‘C’, ‘G’) and include insertions and deletions (indels) in addition to substitutions [1]. These unique features present significant challenges for error prediction, particularly for indels which can shift the reading frame of a sequence, causing errors not only in the affected region but also downstream effects on the entire sequence. To address these challenges, our algorithms take into account the unique complexities of indels in DNA channels involving error prediction model, shift algorithm and CRC64 backtracking.

Due to the high degree of difficulty of predicting true values of substitution and deletion errors in DNA sequences, Derrick uses a strategy to convert as many of them as possible to insertions which are easy to identify and correct afterwards. However, as Fig. S5 shows, a small proportion of substitution and deletion errors still exist and affect the performance of the current version of algorithms. There are two potential solutions. First, DNA synthesis and sequencing technologies, which are able to significantly reduce the proportion of errors other than insertions, need to be developed specially for DDS. Second, a finer prediction model needs to be built so that the true values of substitution and deletion errors can be predicted according to the number of supporting reads for each possible nucleotide type. However, with a limited read coverage, each true value candidate may only be supported by a very small number of reads, which is not enough for accurate prediction. In addition, this method will significantly increase the amount of calculation in the subset searching step, because the prediction set and the number of possible subsets will be both much larger than before.

Accurate error prediction is the cornerstone of the soft-decision strategy and Derrick decoding algorithm. Although previous studies have examined the characteristics of DDS channels [9,27], none are generalizable to a different DDS channel, nor have they integrated these features with coding and decoding. We develop a general prediction model by calculating nucleotide confidences from the numbers of supporting reads during consensus building. The accuracy of error prediction is demonstrated to be above 72% using *in vitro* experiments. In the future, studies and observations can be done to find more error-related patterns and regulations in DNA sequences so that the error prediction step can be carried out more accurately and efficiently. More specifically, a precise and comparatively small set of error predictions and a correct order of them according to confidences are able to reduce the running time of the subset searching algorithm in Derrick and improve the number of data blocks successfully solved within a limited time.

Despite high difficulty, it is essential to discuss the theoretical bound of Derrick's error-correcting capability. In contrast to conventional soft-decision decoding methods such as GMD [28] and Chase [22], commonly used in communication technology, Derrick not only predicts error positions but also corrects errors. As such, theoretically, if given unlimited time, Derrick should be capable of solving an unlimited number of errors, excepting those arising from CRC64 collisions [24]. Thus, theoretically, the failure rate is equivalent to the CRC64 collision probability, which is extremely low. However, we argue that the collision probability may not be a suitable measure of capability, as the failing rate under reasonable runtime and number of backtrack conditions is likely to be much higher in most scenarios. Therefore, all results in our study were obtained under a reasonable runtime constraint (6000 s). While providing a strict theoretical bound may be challenging, we have constructed a thorough analysis and derived the mathematical expressions that describe the upper limit of tolerable base error rate of Derrick (see Supplementary Note 3). Our derivation provides an approximate calculation of the theoretical bound, but it has limitations as a key variable is estimated based on experimental results rather than described by theoretical derivation. Future research could explore more accurate estimation methods to further refine our understanding of the error-correcting capability of soft-decision decoding.

When designing a reliable DDS channel, it is important to select an appropriate coding density and sequencing redundancy based on the data volume and sequencing technology to ensure the reliable storage and readout of data. Supplementary Table 4 provides valuable insights, indicating that for large-scale data storage, such as Exabyte-scale, lower code rates (e.g. RS (255, 209)) are more suitable for protecting data and ensuring accurate recovery using Derrick. Conversely, for smaller-scale data storage, such as Megabyte-scale, higher code rates (e.g. RS (255, 241)) are more appropriate, providing high information density while still allowing for accurate recovery. For ultra-large data volumes, a combination of low and high code rates can be selected by first applying error correction with a lower code rate to the overall dataset, and then dividing it into smaller DDS channels and using a higher code rate for each smaller DDS channel.

Although Derrick was originally designed for RS code, the soft-decision decoding strategy and key steps of Derrick, such as error prediction and subset searching, can be directly applied to other ECCs with algebraic decoding algorithms [29], such as Fountain code [4]. While different ECCs encode data in different forms, their hard-decision decoding algorithms all tolerate limited numbers of errors and fail when the numbers exceed this limit. Since the soft-decision strategy and related algorithmic steps in Derrick aim to reduce the number of errors in the code, they are independent from the code form and can help decoding algorithms of different ECCs succeed. In addition to algebraic decoding algorithms, there is another category of ECCs with probabilistic decoding algorithms [29] which error-corrects to a most probable code on the condition of known information. For these ECCs, the algorithmic steps of Derrick cannot be directly applied. However, the principle of soft-decision decoding strategy can still be useful for improving their accuracy. More specifically, the probabilistic model can be improved by considering the accurately predicted errors and their confidences as prior knowledges.

To conclude, Derrick performs well in both *in vitro* and *in silico* experiments and shows great potential to increase the maximum storage volume of DDS to Brontobyte-scale, although it is designed only for showing the clear advantage of the soft-decision strategy over the traditional hard-decision one rather than beating the state-of-the-art ECC systems which may combine more than one ECC code or technology. We believe that state-of-the-art ECC systems can be significantly improved by introducing our proposed idea, and the soft-decision decoding strategy will gradually replace the traditional hard-decision strategy in future ECC systems.

## METHODS

### Derrick encoding process, sequencing and consensus

Derrick encoding process involves converting the files into oligos, including randomization, encoding with RS codes and CRC64 codes, adding indices and primers, then obtaining the oligos for synthesis. In *in vitro* experiments, an oligo pool comprised of 22 950 300-nt oligos was synthesized by Twist Bioscience company, then carried out Illumina and ONT sequencing [30]. For ONT sequencing, due to the significant difference between the read length and oligo length (300 bp), three oligos were concatenated into a molecule of 1020 bp by Gibson assembly before sequencing. In *in silico* experiments, the Illumina, PacBio CLR and ONT sequencing reads were simulated by software like Art_Illumina [31] and PBSIM2 [32,33]. More details of these steps are shown in Supplementary Note 2.

Finally, the reads were grouped by indices and the consensus sequences were built by performing multiple-sequence-alignments for the reads in the same groups with *bsalign* (https://github.com/ruanjue/bsalign). In addition, the confidence score of each nucleotide was calculated by the number of supporting reads from multiple alignments. Afterward, the consensus sequences of oligos (rows of matrices) were used to regenerate the matrices (Fig. 1A).

### Derrick decoding algorithm

Derrick decoding algorithm, which is the main methodological contribution of this paper, corrects the blocks that original RS hard-decision strategy fails to correct by introducing a soft-decision strategy with a series of steps as follow. First, the algorithm predicts a set of error positions and the corresponding true values as candidates for each RS block. Second, the algorithm takes advantage of the predicted positions and true values to carry out the soft-decision strategy, and makes RS correction succeed. Third, after each RS correction, the algorithm realigns and shifts the columns of the matrix according to the new corrected values to reduce the errors in subsequent blocks. Fourth, after finishing all RS blocks of a matrix, the algorithm checks the overall correctness by CRC64 code, and recursively backtracks the wrong-corrected RS blocks with greedy strategy. The principles and more details of each step are illustrated in Supplementary Note 1 and Supplementary Algorithms 1–4 show the pseudocodes of Derrick decoding algorithm framework.

### Probability of an uncorrectable error

To compare the performances of soft-decision and traditional hard-decision strategies, we calculate the probability of an uncorrectable error (${P}_{UE}$) which represents the probability that the decoding strategy fails to correct a RS block. Letting *E* be the number of errors with known positions and unknown true values (‘erasures’) and *e* be the number of those with unknown positions and true values (‘errors’), the number of unknown variables is *E* + 2*e*. According to RS correction theory, the block fails to be corrected when the number of unknown variables *E* + 2*e* is larger than *n − k*, where *n* and *k* are the total length of code and the length of uncoded original information, respectively. Thus, the ${P}_{UE}$ can be expressed as *P*(*E* + 2*e* > *n* − *k*), where detailed analysis and mathematical calculation are provided in Supplementary Note 3.

## DATA AND CODE AVAILABILITY

The data files containing 19 genomes used for *in vitro* tests were obtained from NCBI Nucleotide with accession numbers shown in Supplementary Table 10. Raw Nanopore sequencing and Illumina sequencing reads for *in vitro* tests had been deposited into the CNCB Genome Sequence Archive (GSA) under GSA accession number CRA008036. The files used for *in silico* tests can be obtained at https://github.com/wushigang2/derrick/tree/main/data_files_insilico/. Derrick source code is hosted by GitHub at: https://github.com/wushigang2/derrick.

## Supplementary Material

nwad229_Supplemental_File
