# Supplementary material for: Improving error-correcting capability in DNA digital storage via soft-decision decoding
Source: Natl Sci Rev. 2023 Sep 2;11(2):nwad229. doi: 10.1093/nsr/nwad229 (PMC10776348; doi:10.1093/nsr/nwad229)
Supplement: nwad229_Supplemental_File [file nwad229_supplemental_file.docx]

Supplementary Materials for

**Improving Error-correcting Capability in DNA Digital Storage via Soft-decision Decoding**

Lulu Ding (丁璐璐)^1,†^, Shigang Wu (伍世刚)^1,†^, Zhihao Hou (侯志浩)^1,2,†^, Alun Li (李阿伦)^1^, Yaping Xu (许亚苹)^1^, Hu Feng (冯虎)^1^, Weihua Pan (潘玮华)^1,*^, Jue Ruan (阮珏)^1,*^

^1^Shenzhen Branch, Guangdong Laboratory of Lingnan Modern Agriculture, Genome Analysis Laboratory of the Ministry of Agriculture and Rural Affairs, Agricultural Genomics Institute at Shenzhen, Chinese Academy of Agricultural Sciences, Shenzhen 518120, China

^2^Guangdong Provincial Key Laboratory of Plant Molecular Breeding, State Key Laboratory for Conservation and Utilization of Subtropical Agro-Bioresources, South China Agricultural University, Guangzhou 510642, Guangdong, China

^*^ Corresponding authors.

E-mails: [ruanjue@caas.cn](mailto:ruanjue@caas.cn), [panweihua@caas.cn](mailto:panweihua@caas.cn).

^†^ These authors contributed equally to this work.

**This PDF file includes:**

Supplementary Note 1 to Note 3

Figs. S1 to S6

Tables S1 to S10

Algorithms S1 to S4

Supplementary Note 1: Derrick decoding algorithm

The Derrick decoding algorithm serves as the primary methodological contribution of this paper. It encompasses four key processes: predicting error positions and true values, applying a soft-decision decoding strategy, implementing a shifting algorithm, and incorporating CRC64 backtracking. The principles and detailed procedures for each step are provided below.

***Predicting error positions and true values.*** The error positions are predicted according to the calculated confidence scores of nucleotides in consensus building step. More specifically, given a RS block with 255 positions (4 nucleotides at each position), a number (e.g., 32) of positions with the lowest confidence scores are chosen as candidate error positions. The confidence score of each position is assigned as the lowest confidence score of the 4 nucleotides at it. For each predicted error position, the algorithm predicts the corresponding true value. Since, in the process of aligning reads into consensus, the algorithm generates comparatively loose and long alignments by adding as many as possible spaces into reads, the majority of errors in consensus sequences are insertions. Thus, at every predicted error position, we assume one of the four nucleotides were inserted by mistake, and removes the one with lowest confidence score. Then the subsequence of this column behind the removed nucleotide shifts to fill the gap and makes a new 4-nucleotide sequence at this position as the true value. At this step, all the operations like removing and shifting are just for predicting true values, but the sequences in matrices are not changed for real.

***Soft-decision decoding strategy*.** The predicted error positions and true values are then used to carry out soft-decision strategy and correct the blocks that traditional RS hard-decision strategy fails to correct. In principle, RS decoder corrects the errors in a given block by solving a system of equations, where the error positions and true values are all unknown variables to be solved. To solve the blocks with number of unknown variables beyond the capability of the system of equations, soft-decision strategy reduces the unknown variables by utilizing the predicted known positions and true values. However, the predicted candidate set of error positions and true values inevitably contains a certain amount of false-positive predictions, and the RS decoder cannot tolerate any incorrect values beyond its capability. Therefore, Derrick uses a searching algorithm that enumerates and provides subsets of the predicted candidate set to the RS decoder one by one, until one with enough correct predictions and without incorrect predictions makes RS decoder succeed or all subsets have been tried.

Let $s (1 \leq s \leq c)$ be the number of error positions selected in the subset, where $c$ is the size of whole prediction set. The algorithm searches iteratively from the subsets containing only 1 error position $(s$ = 1) to the subsets containing all *c* error positions $(s$ = c). For each even $s$, all possible combinations of $s$ error positions are enumerated, and each combination leads to 2 candidate subsets for RS decoder: 1) a subset containing only the *s* error positions which is able to reduce $s$ unknown variables if correct; 2) a subset containing the *s* error positions and the corresponding $s$ true values which are able to reduce 2$s$ unknown variables if correct. For each odd $s$, each combination only leads to 1 candidate subset containing *s* error positions and the corresponding $s$ true values which is able to reduce 2$s$ unknown variables. Since each error corresponds to two unknown variables (position and true value) in RS correction, reducing each 2 variables helps RS decoder solve 1 more error. Therefore, for odd *s*, the subset containing only *s* error positions which is able to reduce $s$ (an odd number) unknown variables and solve only ($s-1)/2$ more errors are seen as meaningless and not tried, because its function has been covered by the subsets with $(s-1$) error positions at previous algorithmic iterations. **Fig. S1** shows an example which describes a simplified process of enumerating subsets.

***Shifting for reducing errors in subsequent blocks.*** After finishing the RS soft-decision correction for one block, Derrick takes advantage of its corrected values to shift the rows of this matrix to reduce the errors in subsequent blocks. First of all, we use an example to explain the shifting process and its purpose (**Fig. S2**). In this example, the sixth nucleotide ‘C’ of the third row is an insertion error which causes errors (e.g., ‘TCGT’, ‘ACGG’) at this row in the second and all subsequent blocks. During RS correction of the second block, ‘TCGT’ at the third row was successfully corrected into ‘TGTA’. Afterwards, for the third row, the corrected subsequence ‘ACAATGTA’ ending at the second block is aligned to the original complete sequence ‘ACAATCGTACGGT…’. Since the last nucleotide ‘A’ of the corrected subsequence is aligned to the ninth nucleotide ‘A’ in original sequence, the nucleotide ‘C’ after ‘A’ is shifted to be the first nucleotide of the third block at this row and the subsequence after ‘C’ is shifted accordingly too. In this way, the subsequent errors (e.g., ‘ACGG’) are corrected (to ‘CGGT’) by one shifting operation, which reduces the number of errors in subsequent blocks and thus the difficulties of their RS corrections.

More formally, after the RS correction of a block where one or more positions have been corrected, the shifting operations are performed on the corresponding rows. When considering one of these rows, denote the original complete sequence as *a*_1_…*a_n_* and the corrected subsequence by now *b_1_*…*b_k_*, the two sequences are aligned by *bsalign*. If *b_k_* is aligned to a nucleotide *a_m_* (1 ≤ *m* ≤ *n*) or a space, the first nucleotide after *a_m_* / the space in *a_1_*…*a_n_* is shifted to become the first nucleotide of the next block at this row, and the subsequence after it is shifted accordingly.

***CRC64 checking and backtracking.*** According to RS correction theory, the blocks with errors are always corrected into a solution of the system of equations which is closest to the original values in space if there are more than one solution (similar with the well-known nearest neighbor method[1]). Although the closest solution is the true value for the majority of blocks, there are ones of which the other solutions such as the second closest or the third closest solution is true, causing wrong corrections. Therefore, after correcting all blocks with RS decoder, Derrick uses CRC64 code to check the correctness of the whole matrix. As long as CRC64 shows the matrix contains one or more error, Derrick backtracks the wrong-corrected blocks and recorrects them by RS decoder.

Here, we propose two backtracking algorithms, namely the backward-searching algorithm and the forward-searching algorithm, both based on a greedy strategy. The backward-searching algorithm is an exact algorithm that can always generate a correct matrix with enough time. Specifically, Derrick searches from the last block in one matrix, and identifies the nearest block which utilizes the soft-decision strategy in the first-round RS correction. Derrick then skips the solutions of this block that have already been tried before and recorrects it using next solution that satisfies the system of equations. Due to the shifting process after each RS correction, the re-correction of one block changes the sequences in subsequent blocks and thus Derrick recursively recorrects all these subsequent blocks. Then the CRC64 checking and the backward searching and re-correction process are carried out iteratively until no errors remain in the matrix.

The forward-searching algorithm is an approximate algorithm which is much faster than backward-searching algorithm and able to generate correct matrix in vast majority of situations. The algorithm searches from the first block and locates the first two continuous blocks that both decoded by soft-decision strategy in the first round RS correction, which is rare and caused by wrong correction of the former block with high probability. Then the algorithm recorrects the former one of the two blocks and all its subsequent blocks in the same way as backward-searching algorithm. Also, the CRC64 checking and the re-correction process are repeated until no errors are detected in the matrix.

In both the backward-searching and forward-searching algorithms, a limit is set on the runtime and backtrack counts for each matrix and the algorithm stops if it is not able to obtain a corrected matrix satisfying CRC64 checking within the time limit or exceeds the backtrack bound. The pseudocodes of the backward-searching and forward-searching algorithms are shown in **Supplementary Algorithm 3** and **4** respectively.

Supplementary Note 2: Derrick encoding process, sequencing and consensus

Although the main methodological innovation of Derrick is about decoding algorithm, to keep the completeness of this paper, we introduce the other parts of Derrick such as encoding process, sequencing and consensus building in this section.

***Encoding.*** The *in vitro* experiments used 19 genomes sequence files and the *in silico* experiments used 6 files of different types such as videos, photos and executables. First of all, the contents in the files were transformed into 0/1 sequences. For the genome sequence file, each nucleotide (A/T/C/G) was converted to the corresponding two bits (00/01/10/11). For other files, each character was read byte by byte and converted to binary. Then the 0/1 sequences were randomized to exclude special DNA patterns such as homopolymers and high GC-content regions[2] which are error-prone in PCR amplification and sequencing[3]. In randomization, instead of carrying out a bitwise exclusive OR operation between the original 0/1 sequence and a pseudorandom 0/1 sequence as the previous works[4,5], Derrick replaced the pseudorandom sequence by the 0/1 sequence of *π* of which the corresponding digit sequence have no apparent pattern and have passed the statistical randomness tests. **Supplementary figure 6** shows that randomness of *π* digit sequence is better than the pseudorandom digit sequence from a widely-used random generator lrand48.

After randomization, RS error-correcting codes and CRC64 codes were added to the original information. **Fig. 1a**). CRC64 code was appended to the end of the matrix in the last column, and RS code was added to the end of each column, after which the matrix was extended to be $n$ rows. In *in silico* and *in vitro* experiments, $l$ and $n$ were always set to be 62 and 255 respectively and $k$ ranged from 211 to 241. Since each entry of the matrix contains 8 continuous bits, each individual row is composed of 496 bits. Afterwards, each individual row was converted into the corresponding DNA sequence by mapping 00/01/10/11 to A/C/G/T.

***Indices and primers addition to DNA sequences.*** In *in vitro* experiments, after encoding the information into DNA sequences, we added indices and primers to the ends of each sequence (**Fig. 1c**). The indices were designed for grouping the sequencing reads from the same sequence (oligo) before building consensus, and the primers were for the downstream PCR-based steps such as Illumina library preparation[6] and Gibson assembly[7].

To generate index sequences, the 0/1 sequence of *π* was transformed into the corresponding nucleotide sequence and partitioned into subsequences of 12 bp. A subset of subsequences satisfying the following rules was then selected as index library: 1) No subsequence containing homopolymers longer than 3 bp; 2) Edit distance between any pair of subsequences larger than 3. Finally, the indices randomly picked from the library were used in the experiments.

Same as indices, the library of primer sequences (20 bp) was generated by partitioning *π* 0/1 sequences too, but satisfied different rules as follows: 1) GC-content between 45% and 55%; 2) No homopolymers longer than 3 bp; 3) No intra-sequence complementary sub-sequence longer than 4 bp; 4) No inter-sequence complementary sub-sequence longer than 8 bp; 5) No common subsequence (≥15 bp) with information sequences or their reverse complement counterparts. For the convenience of downstream Gibson assembly, six pairs of primers were picked from the library for the six original files.

***Oligo synthesis and extraction.*** In *in vitro* experiments, an oligo pool comprised of 22,950 300-nt oligos was synthesized by Twist Bioscience company (South San Francisco, USA). Within this pool, six groups of oligos with equal molar ratio could be distinguished by unique index located at both ends of each oligo. So, each of six could be extracted and transformed to dsDNA by PCR amplification using forward and reverse primers annealing to unique end sequences of oligos, respectively. After receiving the synthetic oligo pool, 1× TE buffer (Invitrogen™, Cat.12090015) was used to dissolved powder. Then each group of oligos were independently extracted by 50 μL PCR reaction comprising following components: 0.8 μL (10 ng) of dissolved oligos served as template, 1.5 μL of both specific forward and reverse primer (10 μM, with length of 20 nt nucleotides), 25 μL of 2× KAPA HiFi HotStart Ready Mix (Roche, Cat.KK2602), and 21.2 μL of nuclease-free water. Then PCR amplification was conducted using a Biometra TOne PCR Thermocycler (Analytik Jena, Germany) according to the following steps: 95 °C for 3 min; 32 cycles of 95 °C for 15 s, 55 °C for 15 s, 68 °C for 20 s; 68 °C for 3 min; 95°C for 3 min and then cooling to room temperature by 0.1 °C s^-1^.

After PCR amplification, six groups of dsDNA products derived from six distinct groups of oligos were evaluated with 2% agarose gel electrophoresis, and gel pieces with the expected 300 bp size were manually cut out and further purified by MinElute Gel Extraction Kit (Qiagen, Cat.28606) according to the manufacturer’s instructions, respectively. The purified DNA concentration of six products were measured using Qubit^TM^ 4.0 fluorometer (Thermo Fisher Scientific, Waltham, MA, USA).

***DNA library preparation and Illumina sequencing.*** Before the library preparation, six products were mixed into one tube with equal molar ratio, and 45 μL mixture (60 ng) was obtained. Then the mixed products were subjected to library preparation and Illumina sequencing with following pipelines:

(1) End-repair and A-tailing. Mix the following components in a sterile nuclease-free tube: 15 μL of End Prep Mix 4 (from VAHTS Universal End preparation Module for Illumina; Vazyme, Cat.N203-01), 45 μL of mixed DNA, and 5 μL of ddH_2_O. After a brief mix and centrifugation, the tube was incubated on a thermal cycler using the following program: heated lid at 65 °C, 15 min at 20 °C, 15 min at 60 °C, and hold at 4 °C.

(2) Adapter ligation. A total of 100 μL ligation reaction comprised following components: 65 μL of End-repair and A-tailing products, 25 μL of Rapid Ligation Buffer 2 (from VAHTS Universal Adapter Ligation Module for Illumina; Vazyme, Cat.N204-01), 5 μL of DNA Adapter, and 5 μL of Rapid DNA Ligase. After a brief mix and centrifugation, the tube was incubated on a thermal cycler, initially for 15 min at 20 °C, and hold at 4 °C. The mixture was purified with 1.8× VAHTS DNA Clean Beads (Vazyme, Cat.N411-01) and eluted with 15 μL sterile ddH_2_O.

(3) Library amplification. After adapter ligation, the products were further amplificated within 50 μL volume comprising following components: 15 μL of ligated product, 5 μL of both universal PPMi5 and PPMi7 primer (10 μM), 25 μL of 2× KAPA HiFi HotStart Ready Mix (Roche, Cat.KK2602). After a brief mix and centrifugation, PCR amplification was conducted according to the following steps: heated lid at 105°C and initial denaturation of 95℃ for 3 min; 8 cycles of 98℃ for 30 s, 60℃ for 30 s, and 72℃ for 30 s; and a final extension at 72℃ for 5 min. Then PCR products were purified with 1.8× VAHTS DNA Clean Beads (Vazyme, Cat.N411-01) and eluted with 20 μL sterile ddH_2_O.

(4) Quality control of library. After library preparation, the concentration and size profile of library were measured by Qubit^TM^ 4.0 Fluorometer (Thermo Fisher Scientific, Waltham, MA, USA) and the Agilent 2100 Bioanalyzer (Agilent Technology, CA, USA), respectively.

(5) Sequencing. Qualified library was sent to Novogene Co., Ltd (Beijing, China) and sequenced by Illumina NovaSeq 6000 sequencer (Illumina, USA) with 250 bp paired-end reads.

***ONT sequencing.*** Given the significant difference between the read length of ONT and amplified dsDNA products (300 bp), three short dsDNA fragments were ligated into a long molecule (with length of 1,020 bp) by Gibson assembly before ONT sequencing.

(1) Overhangs ligation. For each of six groups of amplified dsDNA products (obtained in section of Oligo synthesis and extraction), distinct overhang regions (included in primer) necessary for subsequent Gibson assembly were ligated at the ends of dsDNA by PCR amplification with 50 μL of reaction system: 1 μL of dsDNA PCR products (~1 ng), 1.5 μL of both forward and reverse primer (10 μM, with length of 50 bp), 25 μL of 2× KAPA HiFi HotStart Ready Mix (Roche, Cat.KK2602), and 21 μL of nuclease-free water. PCR amplification was conducted according to the following procedures: 95 °C for 3 min; 32 cycles of 95 °C for 15 s, 60 °C for 15 s, 68 °C for 30 s; 68 °C for 3 min; 95°C for 3 min and then cooling to room temperature by 0.1 °C s^-1^_._ After amplification, the products of six groups were evaluated with 2% agarose gel electrophoresis, and gel pieces with the expected 360 bp size were manually cut out and further purified by MinElute Gel Extraction Kit (Qiagen, Cat.28606), respectively. The concentration of each group of products were determined by Qubit^TM^ 4.0 fluorometer (Thermo Fisher Scientific, Waltham, MA, USA).

(2) Gibson assembly. After purification and quantification, three of six groups of products (Group 1 to Group 3; Group 4 to Group6) were mixed with equal molar ratio, and Gibson assembly was performed using Gibson assembly master mix (NEB, Cat.E2611L) according to the manufacturer’s recommendations.

(3) Longest fragment recovery. After assembly, the longest fragments were amplified with primers annealing to unique overhang sequences presented at the 5′ and 3′ ends of the DNA. PCR reaction included: 1 μL of assembled products (~1 ng), 1.5 μL of both forward and reverse primer (10 μM), 25 μL of 2× KAPA HiFi HotStart Ready Mix (Roche, Cat.KK2602), and 21 μL of nuclease-free water, and then amplification was conducted according to the following procedures: 95 °C for 3 min; 32 cycles of 95 °C for 15 s, 60 °C for 15 s, 68 °C for 1 min; 68 °C for 3 min; 95°C for 3 min and then cooling to room temperature by 0.1 °C s^-1^_._ After amplification, the products were evaluated with 2% agarose gel electrophoresis, and gel pieces with the expected 1000 bp size were manually cut out and further purified by MinElute Gel Extraction Kit (Qiagen, Cat.28606).

(4) Library preparation and ONT sequencing. The purified fragments were delivered to Novogene Co., Ltd (Beijing, China) for long-read genomic library construction and then sequenced using the MinION nanopore sequencer (Oxford Nanopore Technologies, UK). At last, a total of 55 million 1D reads were produced.

***Sequencing simulation for in silico experiments.*** In *in silico* experiments, software was used to simulate sequencing process for each pseudo-oligo. A popular long-read simulator PBSIM2[8] was used to generate ONT and PacBio CLR reads with ‘--nanopore’ and ‘--pacbio’ options respectively. The sequencing depth was set to be 2,000 and the ratio of different types of sequencing errors was 23(substitution):31(insertion):46(deletion). All other parameters were default. Art_Illumina[9] was used to simulate Illumina paired-end reads with parameters ‘-ss MSv1 -p -l 250 -f 1000 -m 300 -s 10’.

***Subsampling.*** For each of ONT, PacBio and Illumina data in *in vitro* or *in silico* experiments, we generated sub-datasets for comparison by randomly subsampling reads with different sequencing depths (ranging from 2× to 10×) from the whole read set.

***Building consensus.*** To build consensus sequences, multiple-sequence-alignment processes were performed to align the reads. In *in silico* experiments, the simulated reads of each pseudo-oligo were directly aligned by *bsalign* (<https://github.com/ruanjue/bsalign>) with parameters ‘poa -L -G’ into consensus sequences. In *in vitro* experiments, the reads were grouped according to indices before generating consensus sequence. More specifically, the Illumina reads with same index were grouped directly, while the ONT reads were partitioned into subreads by primers firstly due to the previous step of Gibson assembly and then the subreads were grouped by index. After grouping the reads or subreads with reliable indices, those containing no index and those containing indices with sequencing errors were assigned to groups they were best sequence-aligned to. Finally, the consensus sequences were generated by *bsalign* in the same way as *in silico* experiments. In addition, *bsalign* calculated a confidence score for each nucleotide on consensus sequence by the number of supporting reads, and the score would be used in Derrick decoding algorithm for predicting errors in consensus.

Afterwards, we arranged the consensus sequences of oligos (rows) back into the matrix (**Fig. 1A**). Due to the indel errors existing, the sequences may have different lengths. To unify the lengths, we aligned the starting nucleotides of sequences (rows) and appended ‘A’s at the ends to a fixed length.

Supplementary Note 3: Probability calculation for evaluating the performance of Derrick

***Calculating the probability of an uncorrectable error.*** To calculate the probability, the probabilistic distribution of ($E+2e)$ for each strategy needs to be estimated first. Following previous research work[10], we assume $E$ and $e$ both be Poisson distributed, and thus $(E+2e)$ is Poisson distributed too due to the additive property. To estimate the parameters (means) of distributions, a set of blocks from real datasets are collected as samples for each RS code framework ($n$ and $k$) and sequencing depth, and the errors of these blocks are identified by comparing with “ground truth” sequences. For hard-decision strategy, all these errors are treated as position-unknown and true-value-unknown ones, and the $P_{UE}$ is degraded into $P(2e>n-k)$ with $E=0$. For soft-decision strategy, although Derrick predicts not only error positions but also true values, here we only consider the errors with two unknown variables (position and value) changing to those with one unknown variable (only value) by position prediction. More specifically, the errors with the positions correctly predicted by Derrick are changed to ‘Erasures’ from ‘errors’. Thus, the Poisson distributions of hard-decision and soft-decision strategies can be estimated and $P_{UE}$ can be calculated respectively. $P_{UE}$ was also called the survivor fraction of Poisson distribution[11]. Then we calculated it by the python function *poisson.sf* from the library of scipy.stats. Finally, the ratio of two $P_{UE}$ is used to evaluated the improvement of soft-decision strategy compared to hard-decision.

***Theoretical bound of error-correcting capability.*** While Derrick has the theoretical capability to solve an unlimited number of errors barring CRC64 collision[12], real-world applications introduce practical constraints that lower this capability. Specifically, the size of the prediction set is curtailed by the acceptable subset-searching runtime. Additionally, even this reduced capability cannot be guaranteed with 100% certainty due to the possibility of wrong corrections in soft-decision decoding. When the number of errors exceeds the error-correcting capability of hard decision decoding, RS decoding may encounter wrong correction. The erroneous block is initially decoded into solutions that closely mirror the correct values but aren't the correct values. Derrick attempts to mitigate this limitation by leveraging backtracking algorithms, but the number of backtracks is also constrained by the tolerable running time. Overall, the probability of unsuccessful error-correction influenced by the acceptable runtime and the number of backtracks. Based on these analyses, we have derived the following mathematical expressions that define the theoretical error-correcting capability of soft-decision decoding, the upper bound of the base-error-rate, and the probability of Derrick not achieving this capability.

First, we analyze the theoretical error-correcting capability of soft-decision decoding on the basis of hard decision decoding capability. For a RS ($n, k$) block, $n$ represents the total length of the RS block in symbols, $k$ represents the length of uncoded original information in symbols, and $(n-k)$ represents the length of redundancy in symbols. With hard-decision decoding, the number of correctable symbol errors $t$ is bounded by:

$$2t\leq n-k \left( 1 \right).$$

Therefore, hard-decision decoding has a definite upper limit of correctable errors: $\frac{n-k}{2}$. With soft-decision decoding strategy which only predicts the error positions, the error-correcting capability is bounded by:

$$E+2e\leq n-k (2)$$

where $E$ represents the number of errors with position predicted and $e$ represents the number of errors with unknown locations. With soft-decision decoding strategy which predicts both error positions and true values, the error-correcting capability is bounded by:

$$2\left( t-c \right)\leq n-k (3)$$

where ‘c’ refers to the number of errors with predicted positions and true values.

Derrick not only predicts the error positions but also corrects errors. However, for a conservative analysis, we utilize the inequality (2) for calculation.

Let ε be the ratio of errors with predicted positions, inequality (2) can be transformed into:

$\varepsilon\times t+2\times(1-\varepsilon)\times t \leq n-k$ (4).

Our experiments have shown that the ratio of errors with predicted positions ε can be estimated to be above 72% (Fig. 2D, Fig. S3A, and Fig. S4). Substituting 72% into inequality (4), we can derive the upper bound of $t$ (the number of correctable symbol errors) as:

$t =[\frac{n-k}{1.28}]$ (5)

and the upper bound of the tolerable symbol error rate as:

$P_{SE}=\frac{t}{n}$ (6).

To further estimate the upper limit of the base error rate, we assume the base errors occur independently, and the relationship between base-error-rate $P_{B}$ and symbol-error-rate $P_{SE}$ is shown as:

$P_{SE}=1-\left( 1-P_{B} \right)^{l}$ (7).

Next, we estimate the probability $P_{D}$ that the upper limit of the base error rate cannot be achieved due to the wrong correction. According to Swanson and Mceliece [13], for a RS(n, k) block, the upper bound of the wrong correction probability of soft-decision decoding can be shown as:

$$P_{E}\left( u \right)\leq n^{-\left( n-k \right)}\sum_{i=n-k+1-u}^{\frac{n-k}{2}} C_{n}^{i}n^{i} (8)$$

where $u$ denotes the number of symbol errors. Since Derrick allows 180,000 (default parameter) backtracks, the upper limit of the base error rate cannot be achieved only when the wrong corrections occur in all of these backtracks. By assuming the independence of these backtracks, $P_{D}$ can be calculated as ${P_{E}\left( u \right)}^{180,000}$. Given the default parameters of Derrick, the value of $P_{D}$ should be negligible, implying that the upper limit of the base error rate (given by equations (4)-(7)) can be achieved with a high probability.

**References**

1. MILOUD-AOUIDATE A and BABA-ALI AR. Survey of Nearest Neighbor Condensing Techniques. *Inter J Adv Comput Sci Appl* 2011; **2**.

2. Erlich Y and Zielinski D. DNA Fountain enables a robust and efficient storage architecture. *Science* 2017; **355**: 950-4.

3. Xu C, Zhao C and Ma B *et al.* Uncertainties in synthetic DNA-based data storage. *Nucleic Acids Res* 2021; **49**: 5451-69.

4. Meiser LC, Antkowiak PL and Koch J *et al.* Reading and writing digital data in DNA. *Nat Protoc* 2020; **15**: 86-101.

5. Organick L, Ang SD and Chen YJ *et al.* Random access in large-scale DNA data storage. *Nat Biotechnol* 2018; **36**: 242-8.

6. Shendure J, Balasubramanian S and Church GM *et al.* DNA sequencing at 40: past, present and future. *Nature* 2017; **550**: 345-53.

7. Gibson DG, Young L and Chuang RY *et al.* Enzymatic assembly of DNA molecules up to several hundred kilobases. *Nat Methods* 2009; **6**: 343-5.

8. Ono Y, Asai K and Hamada M. PBSIM2: a simulator for long-read sequencers with a novel generative model of quality scores. *Bioinformatics* 2021; **37**: 589-95.

9. Huang W, Li L and Myers JR *et al.* ART: a next-generation sequencing read simulator. *Bioinformatics* 2012; **28**: 593-4.

10. Press WH, Hawkins JA and Jones SK Jr *et al.* HEDGES error-correcting code for DNA storage corrects indels and allows sequence constraints. *Proc Natl Acad Sci U S A* 2020; **117**: 18489-96.

11. Klar B. Bounds on tail probabilities of discrete distributions. *Probab Eng Inform Sci* 2000; **14**: 161-71.

12. Jones DT. *An Improved 64-bit Cyclic Redundancy Check for protein sequences*[abstract]. UCL, London, 2009.

13. Swanson L and McEliece R. On the decoder error probability for Reed - Solomon codes. *IEEE Trans Inf Theory* 1986; **32**: 701-3.


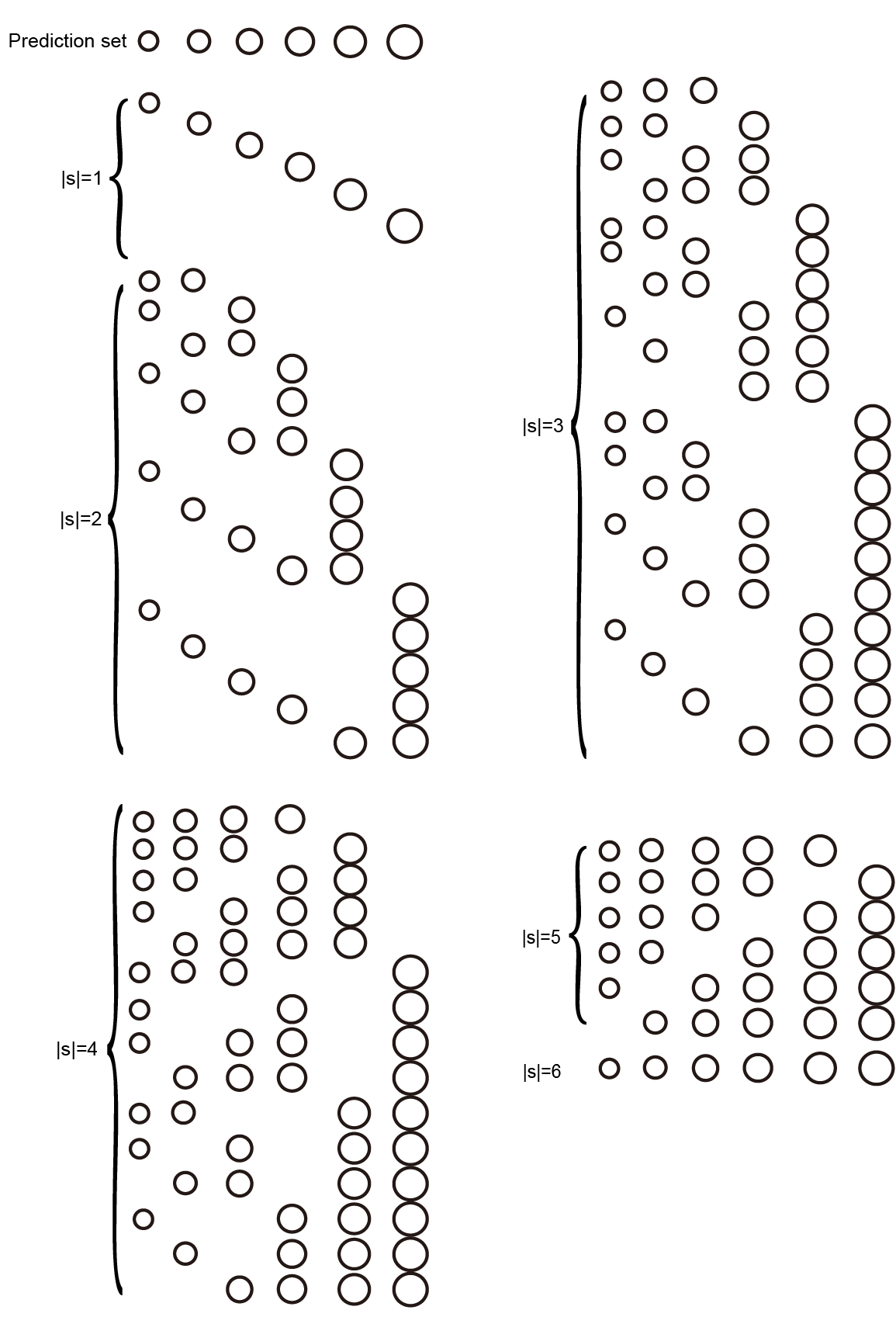


Fig. S1. Illustrating the procedure of selecting subsets of predictions from candidate set in Derrick decoding algorithm. The size of candidate set is assumed to be six in this example; the first row shows the ordered six predictions (represented by circles of different sizes) in candidate set; each of the second to last rows shows one subset selected; *S* represents the subset selected; the order of the subsets selected in Derrick decoding algorithm is shown from top to bottom in the figure.


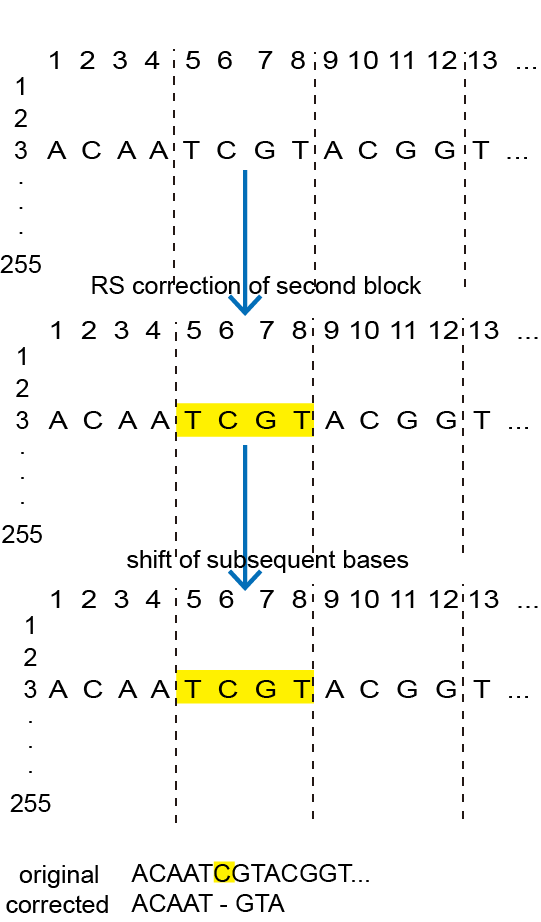


Fig. S2. An example of the shifting step in Derrick decoding algorithm. The whole process of this example is described in Supplementary note 1.


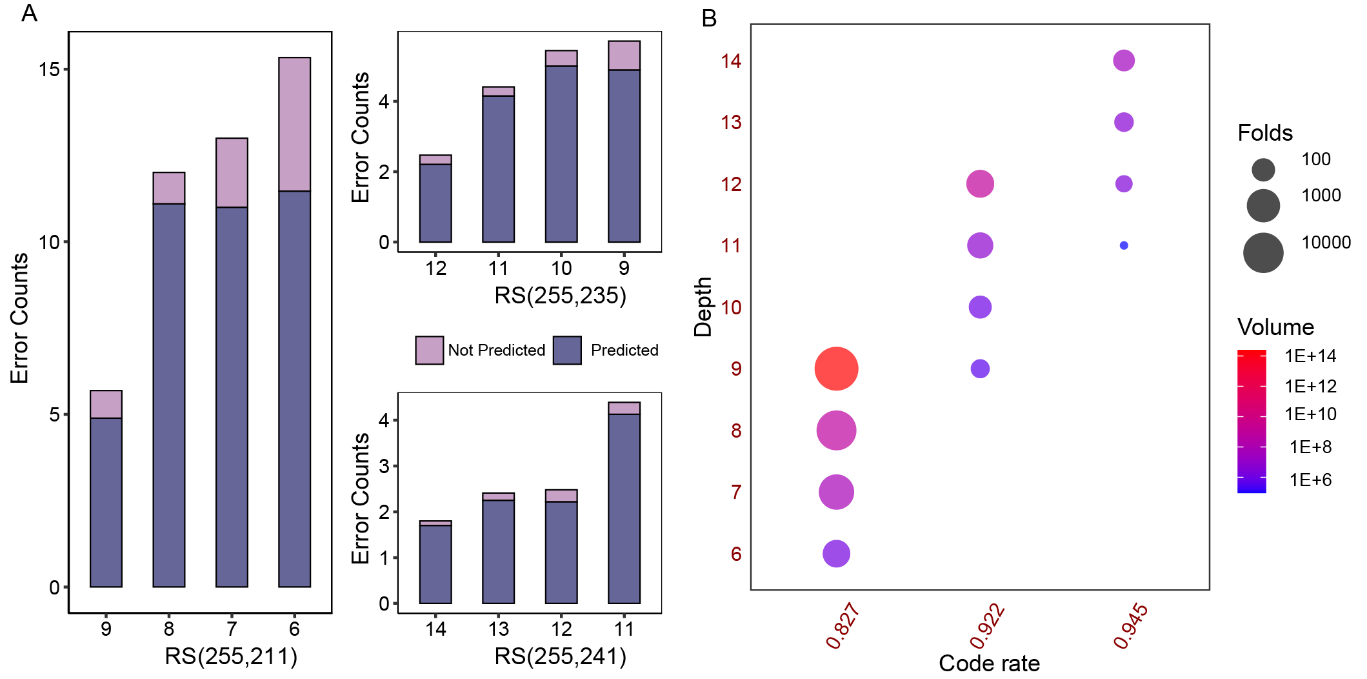


**Fig. S3. Results of *in silico* experiments with ONT sequencing.** (**A**) The prediction performance with RS(255, 211), RS(255, 235) and RS(255, 241). The horizontal axis represents sequencing depth, while the vertical axis represents the number of errors. The dark and shallow colors indicate the number of successfully predicted errors and the number of errors that could not be predicted, respectively. (**B**) The improvement of soft-decision strategy compared with hard-decision one. The horizontal axis represents the code rate, while the vertical axis represents the sequencing depth. The size of the circle represents the folds of improvement, which is the ratio of the probability of an uncorrectable error calculated using the soft-decision strategy versus the probability of an uncorrectable error calculated using the hard-decision strategy. The color of the circle represents the achievable storage volume of soft-decision decoding.


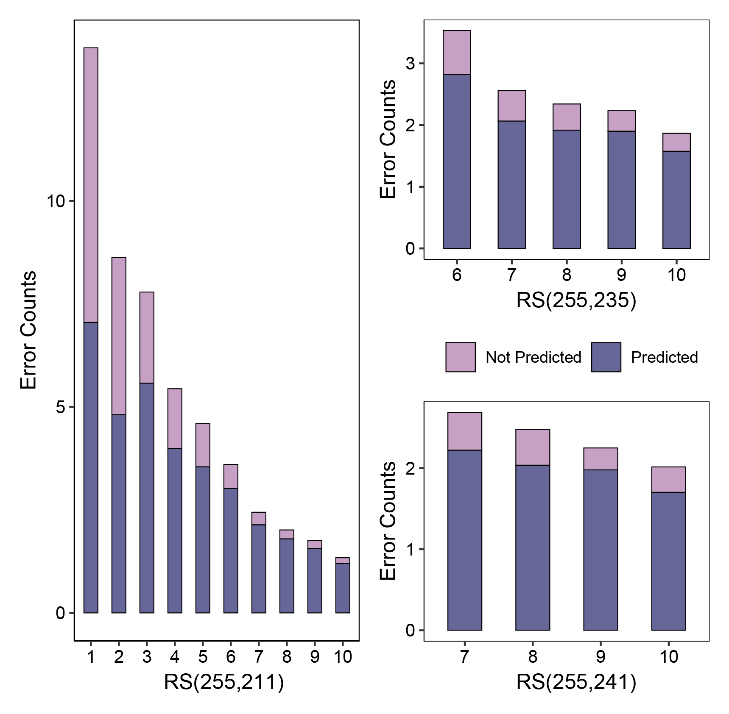


Fig. S4. Results of *in vitro* experiments with Illumina PE250 sequencing prediction performance with RS (255, 211), RS (255, 235) and RS (255, 241). The horizontal axis and vertical axis represent the sequencing depth and number of errors respectively; dark and shallow represent the number of successfully predicted errors and the number of errors failed to predict.


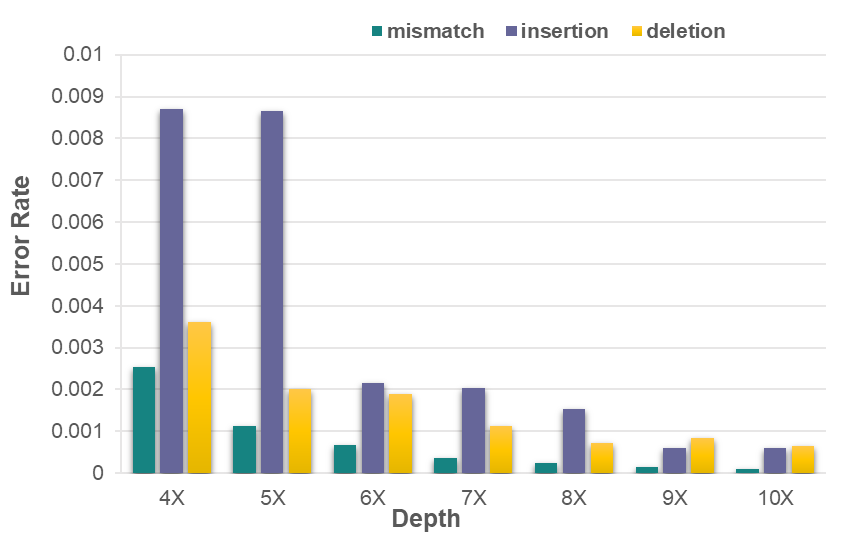


Fig. S5. Error distribution in real ONT consensus sequences. The horizontal axis and vertical axis represent the sequencing depth and error rate of three error types respectively: mismatch, insertion and deletion.


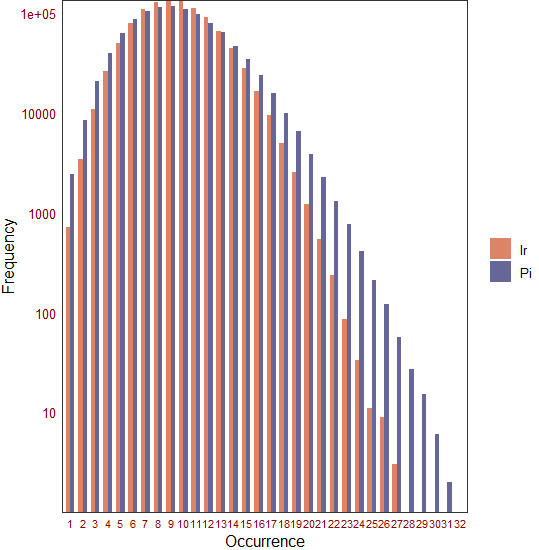


Fig. S6. Comparison of the randomization effects of π binary sequence with the binary sequence from a widely-used C random number generator lrand48(). The two binary sequences were cut into 20-mers (0/1 subsequence of length 20) and 9,999,990 20-mers were collected for each; lr and Pi represent lrand48() and π respectively; horizontal axis and vertical axis represent the number of occurrences and the number of 20-mers for specific number of occurrences respectively; a ‘thinner’ distribution of π 20-mers shows a better randomization effect of π than lrand48().

| **Files Name** | **File size (Bytes)** | **Compressed Size (Bytes)** | **RS (*n, k*)** | **#Oligos** |
| --- | --- | --- | --- | --- |
| **ecolin.k12.MG1655** | 4,641,652 | 1,160,413 | RS (255,235) | 20,400 |
| **Covia-19.8** | 238,944 | 59,620 | RS (255,241) | 1,020 |
| **Covia-19.10** | 307,260 | 76,815 | RS (255,211) | 1,530 |
| **Total** | **5.2MB** | **1.3MB** | **-** | **22,950** |

**A**

| **File Name** | **File Size (Bytes)** |
| --- | --- |
| **pgzf** | 317,450 |
| **group.jpg** | 6,921,338 |
| **playfun.mp4** | 877,291 |
| **wtdbg2** | 1,843,787 |
| **wtdbg-cns** | 825,868 |
| **wtpoa-cns** | 891,097 |
| **Total** | **11,676,831** |

**B**

Table S1. File library for testing Derrick and soft-decision decoding strategy. (a) Files used in *in vitro* experiments; *n* and *k* in RS (*n*, *k*) represent code length and length of uncoded original information respectively. (b) Files (4 executable files, 1 photograph, and 1 video) used in *in silico* experiments.

| **RS (*n, k*)** | **RS (255, 211)** | | **RS (255, 235)** | | **RS (255, 241)** | |
| --- | --- | --- | --- | --- | --- | --- |
| **Size** | 44 | | 32 | | 20 | |
| **#Matrix** | 120 | | 1600 | | 80 | |
| **Depth** | hard | Derrick | hard | Derrick | hard | Derrick |
| **2×** | 30 | 17 | - | - | - | - |
| **3×** | 19 | 5 | - | - | - | - |
| **4×** | 0 | 0 | - | - | - | - |
| **5×** | 0 | 0 | 34 | 1 | 23 | 7 |
| **6×** | 0 | 0 | 3 | 0 | 6 | 1 |
| **7×** | 0 | 0 | 1 | 0 | 2 | 0 |
| **8×** | 0 | 0 | 1 | 0 | 0 | 0 |
| **9×** | 0 | 0 | 0 | 0 | 0 | 0 |
| **10×** | 0 | 0 | 0 | 0 | 0 | 0 |

Table S2. Performance comparison of the Derrick decoding algorithm with hard-decision decoding strategy by the number of failed matrices on Illumina real datasets. The ‘Size’ represents the size of prediction set; ‘#Matrix’ row represents the total number of matrices for each decoding test; ‘Depth’ column represents the sequencing depths; ‘hard’ and ‘Derrick’ represent the number of failed matrices for hard-decision decoding strategy and Derrick decoding algorithm respectively; ‘-’ represents the number of errors in most matrices in the tests exceed the capability of Derrick and the result is not counted.

| **RS (*n, k*)** | **RS (255, 211)** | | **RS (255, 235)** | | **RS (255, 241)** | |
| --- | --- | --- | --- | --- | --- | --- |
| **Size** | 44 | | 32 | | 20 | |
| **#Matrix** | 894 | | 802 | | 782 | |
| **Depth** | hard | Derrick | hard | Derrick | hard | Derrick |
| **6×** | 775 | 2 | - | - | - | - |
| **7×** | 262 | 0 | - | - | - | - |
| **8×** | 136 | 0 | 772 | 20 | - | - |
| **9×** | 0 | 0 | 671 | 2 | - | - |
| **10×** | 0 | 0 | 617 | 2 | - | - |
| **11×** | 0 | 0 | 226 | 0 | 778 | 82 |
| **12×** | 0 | 0 | 3 | 0 | 162 | 0 |
| **13×** | 0 | 0 | 0 | 0 | 151 | 0 |

Table S3. Performance comparison of the Derrick decoding algorithm with hard-decision decoding strategy by the number of failed matrices on ONT simulated datasets. The ‘Size’ represents the size of prediction set; ‘#Matrix’ row represents the total number of matrices for each decoding test; ‘Depth’ column represents the sequencing depths; ‘hard’ and ‘Derrick’ columns represent the number of failed matrices for hard-decision decoding strategy and Derrick decoding algorithm respectively; ‘-’ represents the number of errors in most matrices in the tests exceed the capability of Derrick and the result is not counted.

| **RS (*n, k*)** | **#Matrix** | **Strategy** | **6×** | **7×** | **8×** | **9×** | **10×** | **11×** | **12×** | **13×** | **14×** | **15×** |
| --- | --- | --- | --- | --- | --- | --- | --- | --- | --- | --- | --- | --- |
| **RS (255, 201)** | 938 | hard | 74 | 0 | 1 | 0 | 0 | 0 | 0 | 0 | 0 | 0 |
|  |  | Derrick | 1 | 0 | 0 | 0 | 0 | 0 | 0 | 0 | 0 | 0 |
| **RS (255, 205)** | 920 | hard | 200 | 1 | 14 | 0 | 0 | 0 | 0 | 0 | 0 | 0 |
|  |  | Derrick | 0 | 0 | 0 | 0 | 0 | 0 | 0 | 0 | 0 | 0 |
| **RS (255, 209)** | 902 | hard | 524 | 9 | 46 | 0 | 0 | 0 | 0 | 0 | 0 | 0 |
|  |  | Derrick | 1 | 0 | 0 | 0 | 0 | 0 | 0 | 0 | 0 | 0 |
| **RS (255, 213)** | 885 | hard | 825 | 38 | 219 | 0 | 0 | 0 | 0 | 0 | 0 | 0 |
|  |  | Derrick | 12 | 0 | 0 | 0 | 0 | 0 | 0 | 0 | 0 | 0 |
| **RS (255, 217)** | 869 | hard | 869 | 187 | 589 | 0 | 0 | 0 | 0 | 0 | 0 | 0 |
|  |  | Derrick | 50 | 0 | 2 | 0 | 0 | 0 | 0 | 0 | 0 | 0 |
| **RS (255, 221)** | 853 | hard | 853 | 502 | 828 | 0 | 0 | 0 | 0 | 0 | 0 | 0 |
|  |  | Derrick | 133 | 0 | 14 | 0 | 0 | 0 | 0 | 0 | 0 | 0 |
| **RS (255, 225)** | 838 | hard | - | - | 838 | 1 | 0 | 3 | 0 | 0 | 0 | 0 |
|  |  | Derrick | - | - | 9 | 0 | 0 | 0 | 0 | 0 | 0 | 0 |
| **RS (255, 229)** | 823 | hard | - | - | 823 | 22 | 5 | 27 | 0 | 0 | 0 | 0 |
|  |  | Derrick | - | - | 240 | 0 | 0 | 0 | 0 | 0 | 0 | 0 |
| **RS (255, 233)** | 809 | hard | - | - | - | - | - | 156 | 0 | 0 | 1 | 0 |
|  |  | Derrick | - | - | - | - | - | 0 | 0 | 0 | 0 | 0 |
| **RS (255, 237)** | 796 | hard | - | - | - | - | - | 642 | 6 | 3 | 5 | 0 |
|  |  | Derrick | - | - | - | - | - | 1 | 0 | 0 | 0 | 0 |
| **RS (255, 241)** | 782 | hard | - | - | - | - | - | - | 77 | 56 | 107 | 1 |
|  |  | Derrick | - | - | - | - | - | - | 0 | 0 | 0 | 0 |

Table S4. Performance comparison of the Derrick decoding algorithm with hard-decision decoding strategy by the number of failed matrices on PacBio simulated datasets. The ‘#Matrix’ column represents the total number of matrices for each decoding test; ‘6×’ to ‘15×’ represent the sequencing depths; ‘hard’ and ‘Derrick’ rows represent the numbers of failed matrices for hard-decision decoding strategy and Derrick decoding algorithm respectively; ‘-’ represents the number of errors in most matrices in the tests exceed the capability of Derrick and the result is not counted.

| **RS (*n, k*)** | **RS (255, 211)** | | **RS (255, 235)** | | **RS (255, 241)** | |
| --- | --- | --- | --- | --- | --- | --- |
| **Size** | 44 | | 32 | | 20 | |
| **#Matrix** | 894 | | 802 | | 782 | |
| **Depth** | hard | Derrick | hard | Derrick | hard | Derrick |
| **1×** | 740 | 86 | - | - | - | - |
| **2×** | 0 | 0 | 467 | 364 | - | - |
| **3×** | 0 | 0 | 1 | 0 | 71 | 10 |
| **4×** | 0 | 0 | 1 | 0 | 55 | 8 |

Table S5. Performance comparison of the Derrick decoding algorithm with hard-decision decoding strategy by the number of failed matrices on Illumina simulated datasets. The ‘Size’ represents the size of prediction set; ‘#Matrix’ row represents the total number of matrices for each decoding test; ‘Depth’ column represents the sequencing depths; ‘hard’ and ‘Derrick’ columns represent the number of failed matrices for hard-decision decoding strategy and Derrick decoding algorithm respectively; ‘-’ represents the tests with the number of errors beyond the capability of Derrick and the result is not counted.

| **RS.Depth** | **Size** | **#Failed** | **#Matrix** |
| --- | --- | --- | --- |
| RS211.4× | 20 | 6 | 120 |
|  | 32 | 3 |  |
|  | 44 | 1 |  |
|  | 46 | 1 |  |
|  | 50 | 1 |  |
| RS235.6× | 14 | 104 | 1600 |
|  | 24 | 50 |  |
|  | 32 | 41 |  |
|  | 44 | 48 |  |
| RS241.7× | 14 | 10 | 80 |
|  | 20 | 10 |  |
|  | 32 | 12 |  |

Table S6. Comparison of Derrick decoding algorithm performances with different sizes of prediction set on ONT real datasets. The ‘Size’ represents the size of prediction set; ‘RS211.4×’ represents RS (255, 211) and sequencing depth 4×; ‘RS235.6×’ and ‘RS241.7×’ represent similarly as ‘RS211.4×’; ‘Size’ represents the size of prediction set; ‘#Failed’ represents the number of matrices failed by soft decision strategy; ‘#Matrix’ represents the total number of matrices for each size test.

| **RS (*n, k*)** | **RS (255, 211)** | | | | **RS (255, 235)** | | | | **RS (255, 241)** | | | |
| --- | --- | --- | --- | --- | --- | --- | --- | --- | --- | --- | --- | --- |
| **Depth** | **10s** | **600s** | **6000s** | **Timeout** | **10s** | **600s** | **6000s** | **Timeout** | **10s** | **600s** | **6000s** | **Timeout** |
| **4×** | 106 | 11 | 2 | 1 | - | - | - | - | - | - | - | - |
| **5×** | 117 | 2 | 1 | 0 | - | - | - | - | - | - | - | - |
| **6×** | 120 | 0 | 0 | 0 | 1534 | 11 | 14 | 41 | - | - | - | - |
| **7×** | 120 | 0 | 0 | 0 | 1594 | 3 | 2 | 1 | 60 | 0 | 10 | 10 |
| **8×** | 120 | 0 | 0 | 0 | 1600 | 0 | 0 | 0 | 77 | 0 | 3 | 0 |
| **9×** | 120 | 0 | 0 | 0 | 1600 | 0 | 0 | 0 | 80 | 0 | 0 | 0 |
| **10×** | 120 | 0 | 0 | 0 | 1600 | 0 | 0 | 0 | 80 | 0 | 0 | 0 |

Table S7. Running time (by matrix) distribution of Derrick decoding algorithm on ONT real datasets. On the whole, over 96% of the matrices were corrected successfully within 10 seconds and only 1.6% took more than 6000 seconds; ‘10s’, ‘600s’ and ‘6000s’ columns show the number of matrices finished within corresponding time limits; ‘Depth’ column represents the sequencing depths; ‘Timeout’ columns show the number of matrices not finished within 6000; ‘-’ represents the number of errors in most matrices in the tests exceed the capability of Derrick and the result is not counted.

| **RS (*n, k*)** | **RS (255, 211)** | | | **RS (255, 235)** | | | **RS (255, 241)** | | |
| --- | --- | --- | --- | --- | --- | --- | --- | --- | --- |
| #Matrix | 120 | | | 1600 | | | 80 | | |
| CRC Times | success, crc=1 | success, crc>1 | failure | success, crc=1 | success, crc>1 | failure | success, crc=1 | success, crc>1 | failure |
| **4×** | 117 | 0 | 3 | - | - | - | - | - | - |
| **5×** | 120 | 0 | 0 | - | - | - | - | - | - |
| **6×** | 120 | 0 | 0 | 1525 | 34 | 41 | - | - | - |
| **7×** | 120 | 0 | 0 | 1590 | 9 | 1 | 56 | 12 | 12 |
| **8×** | 120 | 0 | 0 | 1600 | 0 | 0 | 77 | 3 | 0 |
| **9×** | 120 | 0 | 0 | 1600 | 0 | 0 | 80 | 0 | 0 |

Table S8. Statistics of CRC64 code checking on ONT real datasets. The ‘#Matrix’ row represents the total number of matrices for each decoding test; ‘crc = 1’ represents the number of matrices checked by CRC64 code only once and passed detection; ‘crc > 1’ represents the number of matrices failed to decode for the first time and went through backtracking one or more times before success. ‘failure’ represents the number of matrices not successfully decoded within 6,000s; ‘-’ represents the tests with the number of errors beyond the capability of Derrick and the result is not counted.

| **RS(*n, k*)** | **RS(255, 211)** | **RS(255, 235)** | **RS(255, 241)** |
| --- | --- | --- | --- |
| #Matrix | 120 | 1600 | 80 |
| **5×** | 120 | 1600 | 80 |
| **6×** | 120 | 1600 | 80 |
| **7×** | 120 | 1600 | 80 |
| **8×** | 120 | 1600 | 80 |
| **9×** | 120 | 1600 | 80 |
| **10×** | 120 | 1600 | 80 |

depth

**Table S9. Decoding performance for *in vitro* ONT experiments without shifting algorithm.** The values in the table represent the number of failed decoding matrices in the specific RS coding and sequencing depths experiment.

| **File Name** | **Genbank ID** |
| --- | --- |
| Covia-19.10 | MZ544652.1 |
|  | MZ544648.1 |
|  | MZ544655.1 |
|  | MZ544374.1 |
|  | MZ544366.1 |
|  | MZ544367.1 |
|  | MZ410617.1 |
|  | MZ410618.1 |
|  | MZ544651.1 |
|  | MZ544654.1 |
| Covia-19.8 | MZ562973 |
|  | MZ562972 |
|  | MZ562762 |
|  | MZ562761 |
|  | MZ562760 |
|  | MZ562759 |
|  | MZ562758 |
|  | MZ562757 |
| ecolin.k12.MG1655 | NC_000913.3 |

Table S10. The files for *in vitro* tests contain 19 genomes which were obtained from NCBI Nucleotide with accession numbers.


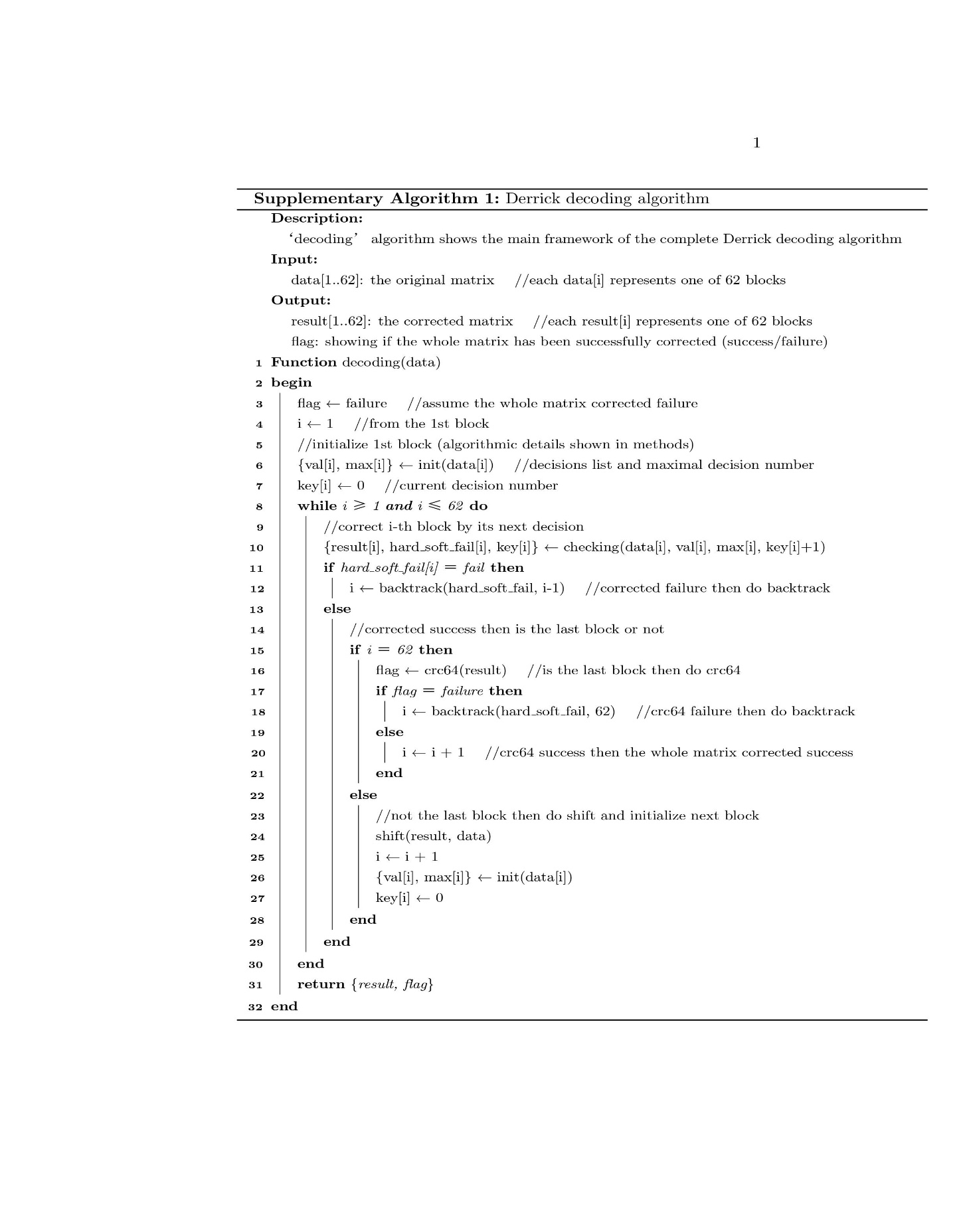


**Algorithm S1. The pseudocode of overall Derrick decoding algorithm.**


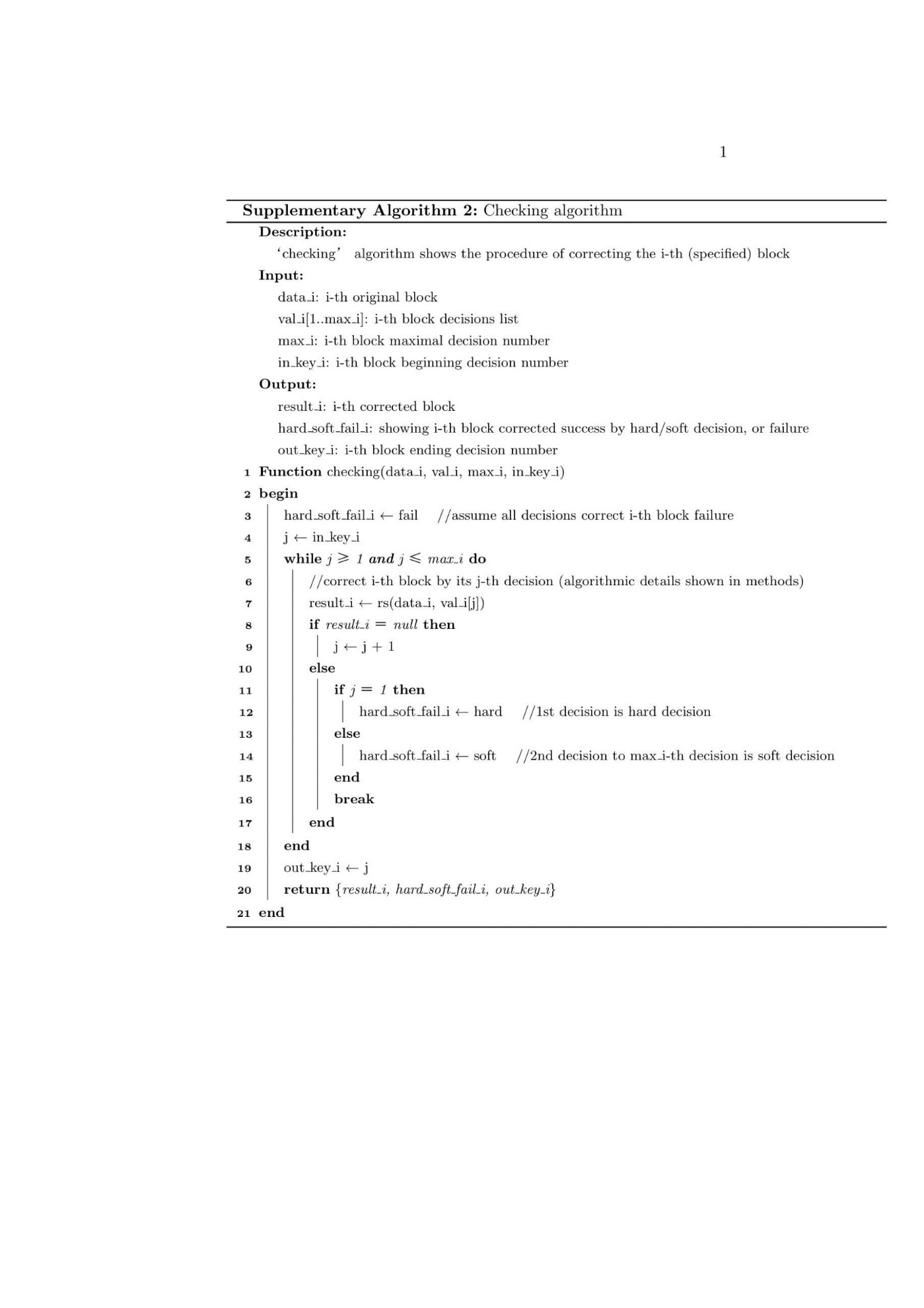


**Algorithm S2. The pseudocode of Derrick checking algorithm.** This algorithm is to decode a specific block.


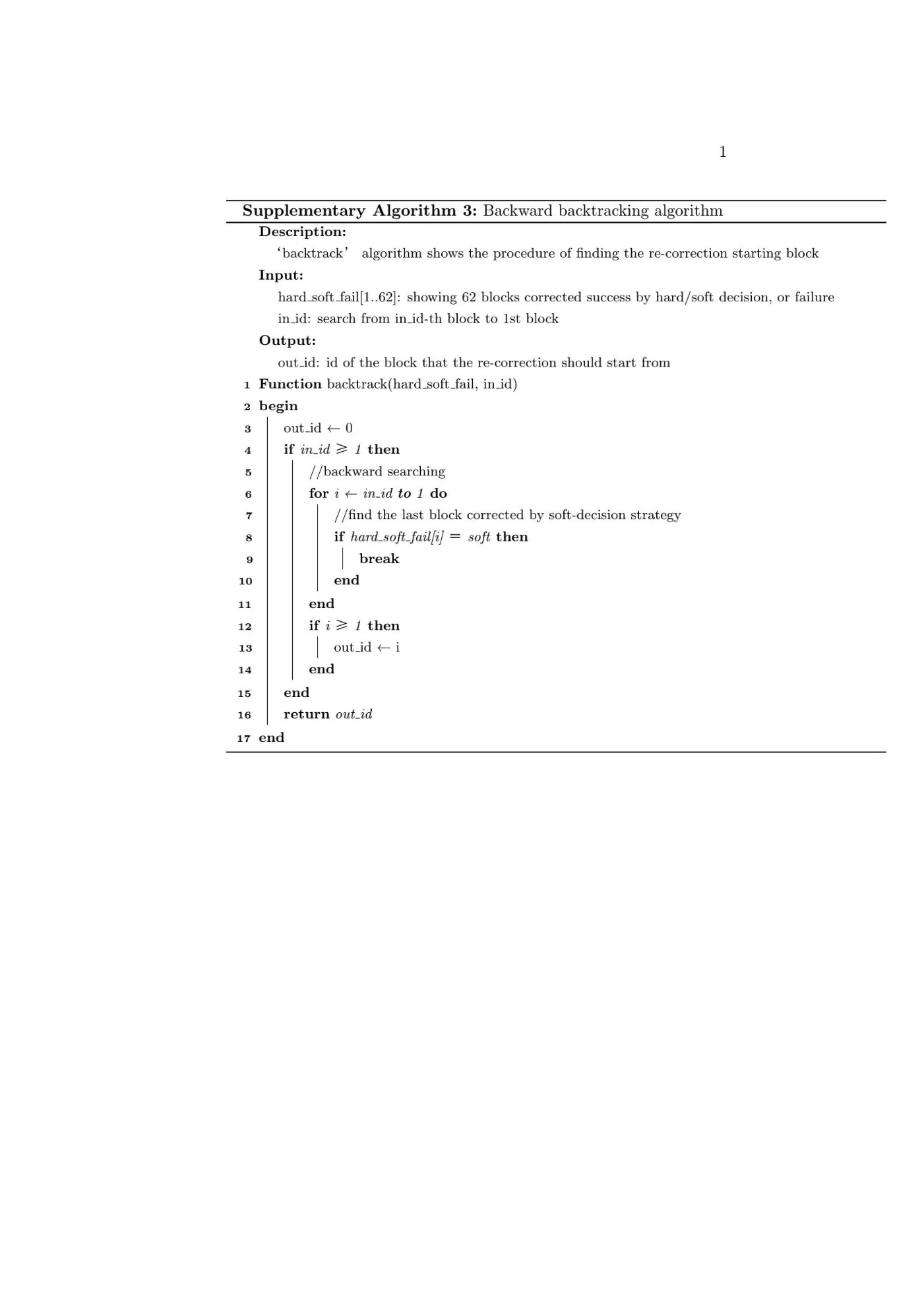


**Algorithm S3. The pseudocode of Derrick Backward backtracking algorithm.** This algorithm aims to search for the occurrence of collision in soft decision decoding among blocks, starting from the last block and progressing towards the first.


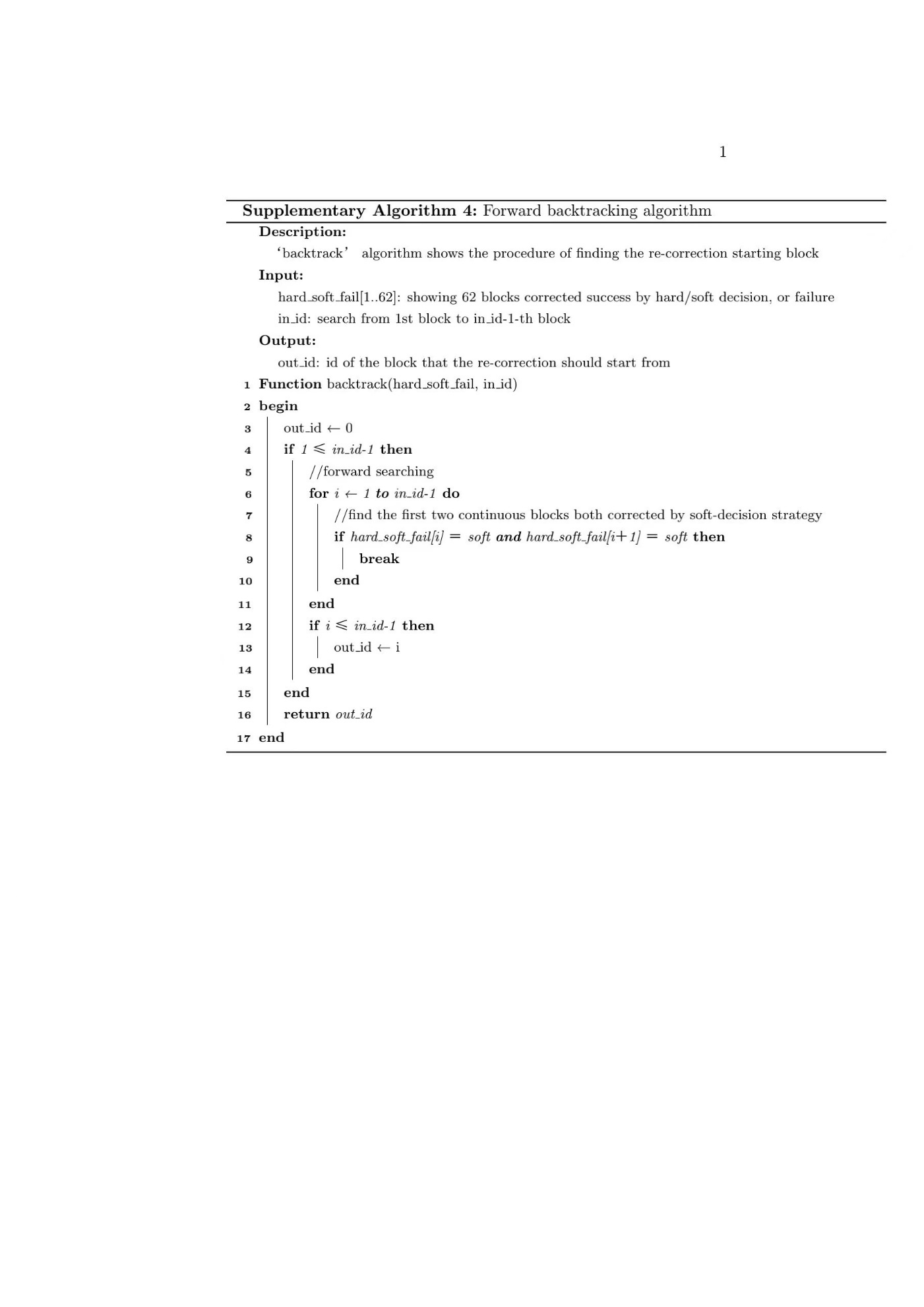


**Algorithm S4. The pseudocode of Derrick Forward backtracking algorithm.** This algorithm aims to search for the occurrence of collision in soft decision decoding among blocks within a matrix, starting from the first block and proceeding towards the last block.
